# A Comprehensive Framework for Uncovering Non-Linearity and Chaos in Financial Markets: Empirical Evidence for Four Major Stock Market Indices

**DOI:** 10.3390/e22121435

**Published:** 2020-12-18

**Authors:** Lucia Inglada-Perez

**Affiliations:** Department of Statistics and Operational Research, Complutense University, Plaza Ramón y Cajal, s/n Ciudad Universitaria, 28040 Madrid, Spain; lucia.inglada.perez@ucm.es

**Keywords:** nonlinear dynamics, chaos, time series analysis, stock exchange market, Lyapunov, recurrence plots, BDS, correlation dimension, GARCH model

## Abstract

The presence of chaos in the financial markets has been the subject of a great number of studies, but the results have been contradictory and inconclusive. This research tests for the existence of nonlinear patterns and chaotic nature in four major stock market indices: namely Dow Jones Industrial Average, Ibex 35, Nasdaq-100 and Nikkei 225. To this end, a comprehensive framework has been adopted encompassing a wide range of techniques and the most suitable methods for the analysis of noisy time series. By using daily closing values from January 1992 to July 2013, this study employs twelve techniques and tools of which five are specific to detecting chaos. The findings show no clear evidence of chaos, suggesting that the behavior of financial markets is nonlinear and stochastic.

## 1. Introduction

Research on modeling financial time series has traditionally assumed linear patterns. Unfortunately, these models are incapable of explaining specific phenomena or market events, such as bubbles and recessions. Therefore, there is interest in and a need to introduce alternative methods, most of which come from other scientific disciplines such as mathematics, physics and engineering [1], to model the dynamics of financial series, and to detect a possible nonlinear and determinist chaotic behavior. This opens up the range of alternatives for analyzing and predicting financial series and particularly stock market price series, traditionally anchored in methods such as technical and fundamental analysis or in assumptions like the efficient market, defined by Fama [2] and based on the fact that asset prices reflect all available information, causing asset returns to be unpredictable [3].

Many scholars have empirically studied the existence of nonlinear dynamics in financial series [4], meanwhile several theoretical models consistent with the presence of nonlinearity in asset prices have arisen [5]. The nonlinear approach can capture the characteristics of the financial series and their sudden fluctuations, and, therefore, plays an important role in economic modeling [6,7]. The initial interest in the application of nonlinear models has been extended to solve the question of whether the nonlinear dynamics have a stochastic or deterministic behavior. This issue constitutes a key point in the process of modeling and forecasting financial time series. The emergence of new models that deal with the volatility present in financial time series, such as the autoregressive conditional heteroskedasticity ARCH [8], generalized (GARCH) [9] and exponential GARCH (EGARCH) [10] models, in consonance with the development of the chaos theory that can explain the effect of shocks on the stock markets as part of the endogenous dynamics of the series itself, constitute a fundamental contribution to solving this issue.

Although the literature on the evidence of chaos in economic and financial time series is extensive, there are no incontrovertible results [11]. There are two divergent positions concerning the underlying dynamics of financial markets. Whilst several studies have found evidence of chaotic patterns in financial markets e.g., [12,13], the most recent studies support a non-chaotic but rather stochastic behavior. This is due to the use of an incorrect specification and to the approach applied [14,15]. Indeed, the former studies applied a methodology, such as the correlation dimension method, that is not sensitive to noise, an intrinsic feature of financial series. However, studies that have used new and more robust methods, such as the test proposed by Matilla-Garcia and Ruiz Marin (MGRM test) [6] and the Lyapunov test based on BenSaïda and Litimi [14], do not find evidence for chaos. In fact, to ensure that conclusions are sound and reliable, traditional tests should be applied to time series that consist of many observations and are noise-free.

The existence of chaos in the stock market remains a relevant issue. Its importance lies in the fact that finding the chaos of low dimension could allow a reliable forecast in the short term, but not in the medium and long term, since a chaotic system is unstable [16]. However, there is no standard methodology for the analysis of the presence of nonlinearity and the existence of a chaotic pattern. Therefore, it is mandatory to explore the existence of a chaotic deterministic structure considering different approaches [17].

The main goal of this research is to shed light on the behavior of stock markets by examining their dynamics and volatility. Through a comprehensive methodological approach, using a wide range of procedures, this study investigates the existence of deterministic chaos and nonlinearity in a series of daily stock indexes of four major stock markets: namely the Dow Jones Industrial Average, the Nasdaq-100, the Ibex 35 and the Nikkei 225. In addition, the paper examines other issues such as the existence of volatility clustering. By using daily closing values from January 1992 to July 2013, this study employs twelve techniques and tools of which five are specific to detecting chaos.

Overall, this work contributes to filling the gap in the international literature on nonlinear and chaotic behavior in stock markets as it uses a variety of tools including the most powerful methods to uncover the underlying dynamics of these markets. Even more so, a review of the international literature (see following section) shows that there are numerous works that aim to detect the existence of nonlinear and chaotic behavior in stock markets, but to the best of our knowledge none of them apply such a high number of techniques as this research.

The methodology applied consisted of the following steps. First, all linear dependencies were removed from the data by applying autoregressive integrated moving average (ARIMA) filters. A wide range of procedures were then applied to the residuals obtained to detect the existence of nonlinearity. If nonlinear dependence was detected, since linear structures had already been removed using the best fit ARIMA model, it was indicative of some type of nonlinear dependence in the returns series resulting from a nonlinear stochastic system or a nonlinear chaotic system. Then it might be caused by the existence of a volatility cluster. In this case, the appropriate generalized autoregressive conditional heteroskedasticity (GARCH) and exponential GARCH models (EGARCH) were applied. Afterwards, the existence of chaotic motion was explored by means of five techniques i.e., 0/1 test, the correlation dimension, Lyapunov exponent, MGRM test [6] and recurrence plots, including those that are ideally suitable for noisy time series analysis.

Empirical findings suggested that although there has been found a dominant nonlinear structure in financial markets, determinism cannot be assumed and hence chaos cannot be inferred.

The rest of the paper is organized as follows: in the second section, a review of the international literature on stock market modelling is carried out, with special emphasis on those works that contrast the possible existence of nonlinear and chaotic behavior. The third section describes the methodological process adopted in this research. In the fourth section, the data sources used as well as the main results obtained for the four stock market index series considered are presented and commented on, and the existing evidence for and against the hypotheses of nonlinearity and a chaotic regime is analyzed and discussed. Finally, the last section draws a series of conclusions on the research carried out and indicates possible future lines of research.

## 2. Literature Review

When studying the existing literature on research aimed at analyzing and contrasting the existence of nonlinear and chaotic dynamics in the stock markets, a first conclusion is that the results obtained depend on the data and methods used in each study. As shown in Table 1, most studies, especially since the appearance of the BDS test [18], suggest the existence of nonlinear dependencies in stock markets.

Whether this nonlinear dependence is from chaotic deterministic origin, a sample of the most representative publications on this issue (Table 1) leads to conclude that it is not possible to give a single answer to this question, although there is a clear tendency in the most recent research towards the alternative of no chaotic behavior in the stock market series. Thus, in the first stage, initiated by the work of Scheinkman and LeBaron [12], who, using daily and weekly data of returns, found evidence of chaotic dynamics in the US stock markets, works that obtained a chaotic behavior were frequent. However, more recently, once new methods and more powerful tests on the detection of chaotic dynamics were incorporated, it can be observed that most research suggests that the data are not chaotic, that is, they present stochastic behavior.

In this regard, many of the studies carried out do not reach a categorical conclusion on the presence of chaos in financial markets due to an erroneous specification of the methods and tests used [14,15]. In fact, the traditional tests used should be applied to time series with a high number of observations and free of noise in order to obtain unambiguous conclusions, but the latter is not usually the case in financial time series, and in particular in stock markets [14,15].

In relation to the development of new tests to detect chaotic behavior, among others, BenSaïda and Litimi [14] incorporated a new test that has been shown to be more powerful in detecting chaos than the method based on the Lyapunov exponent sign, very frequently used in previous studies. Using this test, they analyzed the behavior of ten financial series of returns and concluded that there is nonlinearity in the examined series but in none of them is there a chaotic behavior, suggesting that the chosen financial series are stochastic. Matilla-Garcia and Ruiz Marin [6] proposed a new test to determine whether the dynamics of a series is deterministic (including low dimensional chaos) rather than stochastic and applied it to the daily series of the Dow Jones index. 

## 3. Methodology

### 3.1. Methodological Framework

For the study of the existence of nonlinearity and chaotic behavior, a comprehensive methodological framework has been adopted. Table 2 presents all the techniques used in this research. Their main characteristics are described below, with special emphasis on the tools for detecting chaotic dynamics in a time series.

Before proceeding with the description of the methodological process, it is appropriate to define the concepts of linear and nonlinear series. A stochastic process ($X_{t},\;t\in Z$) is said to be a linear process if for every t∈Z $X_{t}=\overset{\infty }{\underset{j=0}{\sum }}\beta_{j}\epsilon_{t-j}$ where $a_{0}$ = 1, $\epsilon_{t},\;t\in Z$ is a process of independent and identically-distributed random variables (iid) with $E\left[X_{t}\right]=0,\;E\left[X_{t}^{2}\right]=\sigma^{2}$ and $\overset{\infty }{\underset{j=0}{\sum }}\left|\beta_{j}\right|<\infty$. Any process that does not satisfy this condition is said to be nonlinear. Nonlinear models display features that cannot be modelled by linear processes: e.g., higher-moment structures, time-changing variance, volatility clustering, breaks, thresholds, and asymmetric cycles. These traits are very often present in the study of financial time series.

MGRM stands for the test proposed by Matilla-Garcia and Ruiz Marin ([6] Source: Own work)

The methodological framework includes the following steps.

(i) Stationarity testing

Stationarity was studied by means of the following tests:

- Augmented Dickey Fuller Test (ADF)
- Phillips–Perron Test (PP)
- Kwiatkowski–Phillips–Schmidt–Shin Test (KPSS)

After testing that each series under study was not stationary, the log-returns (from now on returns) from each series were obtained by means of the expression: $\mathrm{Ln}\left(R_{t-1}\right)=\ln\left(P_{t}\right)-\ln\left(P_{t-1}\right)$ such that $P_{t}$ is the time series data at time t. 

This new series called “return” has important properties such as stationarity. This property is essential, since it is a prerequisite in a large percentage of both the modelling techniques and the analysis of nonlinearity or chaotic behavior that will be used in this research.

(ii) Linearity modelling

Subsequently, all linear dependence is removed from the return series by applying autoregressive moving average (ARMA) filters. The choice of the model was made as previously described [10], which basically consists of choosing the ARMA (p,q) model which presents the lowest value according to the Schwarz Information Criterion [49]. 

Following the process described by Barkoulas et al. [50], the residuals have been studied. In the case that the residuals were correlated, the order of the ARMA model has been increased, increasing the magnitude of p and q. Once the optimal linear model has been determined, the residuals are extracted from it. The new residuals are called the ARMA series. 

(iii) Nonlinearity testing

The presence of nonlinearity is a necessary but not sufficient condition for the existence of a chaotic deterministic component in the system. Moreover, it is an inherent characteristic of complex systems. To test the existence of nonlinearity and randomness, a wide range of methods has been used, thus managing to supplement the possible limitations of each of them. The following techniques were applied (Table 2).

- The Runs test [39] to evaluate the randomness of the series.
- Keenan’s test [40], a Portmanteau’s test that contrasts the hypothesis of a linear model versus a nonlinear one.
- Tsay’s test [41], which assumes a generalization of the previous test.
- The test of Teräsvirta [42], which is based on the methodology of neural networks.
- White’s test [43], which is also based on the neural network methodology.
- BDS [18,44], one of the most powerful tests for nonlinearity and other types of data dependence.
- Kaplan’s test [5] that has also demonstrated statistical power in determining whether data are linear.

The properties of the tests used in this research are well known in the literature so only the main differences between them are described below.

Keenan’s test [40] examines the hypothesis of a linear model versus a nonlinear one. It is a particular case of the RESET test. The starting hypothesis for the RESET test is that data correspond to a linear model versus the alternative hypothesis that the model is nonlinear. It is based on the idea that if the residuals from the linear model are independent, they should not be correlated with the regressors used in the estimated equation or with the adjusted values and therefore, the regression in the residuals of these values should not be statistically significant. The null hypothesis of linearity is rejected if the value of the F-statistic for the chosen sample exceeds the theoretical value of the F-standard. The RESET test is easy to implement and does not require fitting many parameters and also has a reasonable power to detect some types of nonlinearity.

Keenan [40] proposes a nonlinearity test changing the RESET test to avoid multi-collinearity. Specifically, Keenan assumes that every stationary time series $\left\{Y_{t}\right\}\;$ can be approximated (Volterra expansion) as follows:

(1) $$Y_{t}=\mu +\sum_{u=-\infty }^{\infty }\theta_{u}\varepsilon_{t-u}+\sum_{u=-\infty }^{\infty }\sum_{v=-\infty }^{\infty }\theta_{uv}\varepsilon_{t-u}\varepsilon_{t-v\;\;}$$

such that *μ* is the mean of $Y_{t}$ and $\left\{\varepsilon_{t}\right\}$ is a stationary, independent and identically distributed sequence with a mean of zero. The process is linear, if the term containing the double sum $\overset{\infty }{\underset{u=-\infty }{\sum }}\overset{\infty }{\underset{v=-\infty }{\sum }}\theta_{uv}\varepsilon_{t-u}\varepsilon_{t-v}\;$ is null, that is if all the coefficients *θ* are zero. The method consists of testing whether the coefficient of the double-sum term is zero, to affirm whether the process is linear or not. The test statistic has as a null hypothesis that the time series is linear, and is asymptotically distributed as  F(1, n−2p−2) distribution, where *n* is the sample size, and *p* is the order of linear AR(p) model, that is the number of lags involved in the regression.

The Tsay’s test [41] is a generalization of the previous test. Tsay’s test improved on the power of the Keenan’s test by allowing for disaggregated nonlinear variables, thus generalizing Keenan’s test by explicitly looking for quadratic serial dependence in the data. Tsay [41] showed that corresponding test statistic is asymptotically distributed as Snedecor’s F distribution with the following degrees of freedom: F(m, n−p−m−1) where *n* is the sample size, *m = p(p − 1)/2*, and *p* is the order of AR fitted model, that is the number of lags involved in the regression.

The Teräsvirta test [42] is constructed using neural network models. This test models the original series by means of a Taylor series. It assumes a model of the shape:

(2) $$y_{t}=y_{t}\beta +\sum_{j=1}^{q}\sum_{j=1}^{q}\delta_{j}y_{t-i}y_{t-j}+\sum_{j=1}^{q}\sum_{j=1}^{q}\sum_{j=1}^{q}\delta_{i,j,k}y_{t-i}y_{t-j}y_{t-k}+\varepsilon_{t}$$

The null hypothesis is the following: $H_{0}=\delta_{j}=\delta_{ijk}=0.$

The contrast, and therefore the choice of the optimal model, is made by means of a statistic similar to that used in the previous tests and which attends to an $F\left(p_{2}-p_{1},\;n-p_{2}\right)$ distribution, where $p_{1}$, $p_{2}$ and n are the number of parameters of the first model, the number of parameters of the second model and the sum of both, respectively. 

White’s test [43] also uses neural net methods to test for nonlinearity. In this test, the time series is fitted by a single hidden-layer feed-forward neural network, which is used to determine whether any nonlinear structure remains in the residuals of an AR(p) process fitted to the same time series. White’s test has power to test against various types of nonlinearity in the mean, thus it can be used to distinguish among those nonlinear processes that are nonlinear in the mean and those that are not (such as ARCH and GARCH) [51]. The null hypothesis for the test is linearity in the mean. This methodology has the advantage that a pre-filtering of the conditional variance is unnecessary. A fitted neural net is used to produce the measurable function of the process’s history and an AR(p) process as the linear filter. The hypothesis that the fitted function does not correlate with the residuals of the AR(p) process is then tested. The resulting test statistic has an asymptotic chi squared distribution under the null of linearity in the mean.

BDS [18,44] is one of the most powerful tests for nonlinearity and other types of data dependence. The test uses the correlation integral, a measure of the number of times that temporal patterns are repeated in the data as the test statistic. It was originally formulated to study independence and nonlinear structure (iid) in a time series. However, the test also has statistical power to detect a large number of linear and nonlinear processes. In particular, the authors of the method showed that when applying the test to the residuals of a linear model, if the test rejects the initial hypothesis (i.e., they are iid) it will indicate the existence of linear dependence in the data. Consider a time series ${\left\{y_{t}\right\}}_{t=1}^{T}$ and define its *m*-history as $y_{t}^{m}=\left(y_{t},y_{t-1,}\ldots ,\;y_{t-\left(m-1\right)}\right)$. The BDS tests the null hypothesis that the variable of interest is independently and identically distributed (iid). Under the null hypothesis, the BDS statistic is obtained by

(3) $$V\left(T,m,\varepsilon \right)=\sqrt{T}\frac{C\left(T,m,\varepsilon \right)-C{\left(T,1,\varepsilon \right)}^{m}}{\hat{\sigma }\left(T,m,\varepsilon \right)}$$

The correlation integral asymptotically follows standard normal distribution. $\hat{\sigma }\left(T,m,\varepsilon \right)\;$ is the standard sample deviation of $C\left(T,m,\varepsilon \right)-C{\left(T,1,\varepsilon \right)}^{m}$. Moving from the hypothesis that a time series is IID, the BDS tests the null hypothesis that $C\left(T,m,\varepsilon \right)=C{\left(T,1,\varepsilon \right)}^{m}$, which is equivalent to the null hypothesis of iid against an unspecified alternative [11].

Kaplan’s test [51] has demonstrated statistical power in determining whether data are linear. This test is based on the concept of continuity in deterministic systems, which establishes that after an iteration, two points that are initially close will remain close, while if the underlying model is stochastic two points initially close, they may have images that are very distant from each other. The null hypothesis of the test is the linearity of the system versus the nonlinearity. Kaplan’s test can be used to test either for nonlinearity or for more focused special cases of nonlinearity [51]. In a general and summarized way, given a vector $y_{t}\equiv \left(y_{t},y_{t-\tau ,}\ldots ,\;y_{t-\left(m-1\right)\tau }\right)$ embedded in an m-dimensional phase space and obtained from the set of observed data ${\left\{y_{t}\right\}}_{t=1}^{T}$ and be the image of the point $y_{t}$ for an fixed positive integer named delay τ, $y_{t+\tau }=f\left(y_{t}\right).$

Kaplan’s technique examines for an embedding dimension m and a delay τ all the distances between two pairs of points $\delta_{i,j}=\left|y_{j}-y_{k}\right|$ and their respective images, $\varepsilon_{i,j}=\left|y_{j+\tau }-y_{k+\tau }\right|\;$ to later calculate the average of the values of $\varepsilon_{j,k}$, conditioned to the corresponding value of $\delta_{i,j},\;$ that is $E\left(r\right)\equiv \bar{\varepsilon_{j,k}}\;$ for j and k such that $\delta_{j,k}<r$.

E(r) is therefore an average of all the images, whose initial points are very close. For every deterministic system it is obvious that $\underset{r\to 0}{\lim}E\left(r\right)\to 0$, but for chaotic systems this convergence is not so clear. It is defined then the so-called Kaplan’s (K) statistic as: $K=\underset{r\to 0}{\lim}E\left(r\right)$.

The value of K is expected to be higher in non-deterministic systems, compared to deterministic systems. As mentioned, the null hypothesis of the test is that the underlying model of the data is a linear dynamic system. To perform the test, it needs to work with a K statistic that is calculated for the original series (K test) and a stochastic linear generating model with the same properties (with the same histogram and autocorrelation function) as the original system (KS). If the value of the statistic of the original series exceeds that of the series under the alternative hypothesis of stochastic linearity, the null hypothesis of linearity is accepted. The distribution of the statistic is not tabulated, but Kaplan proposes two maximum levels for testing the null hypothesis of linearity. The first is the minimum KS estimated from the subrogated series, and the second is the mean minus two or three times the standard deviation of all the estimated KS. The null hypothesis of linearity is rejected when the K value calculated for the original reconstructed series is greater than at least one of the two dimensions (called, for simplicity, KS and KSmin respectively).

Despite its simplicity, Kaplan’s test has shown to be able to detect a wide spectrum of nonlinearity classes and has also demonstrated statistical power in determining whether data are linear [51].

(iv) Volatility modelling

A wide range of procedures are then applied to the residuals of the selected ARMA model obtained to detect the existence of nonlinearity. If nonlinear dependence is detected, then it might be caused by the existence of a volatility cluster. This being the case, the conditional variance is modeled by fitting ARCH family models (GARCH and EGARCH), using the same method as above to select the best model. After the estimation of both models, the residuals obtained were standardized by means of their conditional standard deviations (from now on GARCH and EGARCH series).

(v) Study of chaotic behavior

Finally, the existence of the chaotic component in each series was studied. To do this, the following methods were applied (Table 2).

- The Correlation Dimension, a measure of the complexity of a dynamic system that allows a deterministic system to be distinguished from a stochastic one [47].
- Lyapunov’s Exponent, which analyzes in the system the property of sensitivity to initial conditions [14].
- The 0/1 test [46] that provides a value close to zero or one, indicating the latter value existence of chaos in the series.
- MGRM test [6], which has the main advantage of not needing to build the attractor to assess the existence of chaos.
- The Recurrence Plots, which is a visual tool that allows to analyze the existence of periodic patterns, among other aspects of the time series [48].

### 3.2. Tools to Detect Chaotic Regime

To carry out many of the techniques used in this research it is necessary to reconstruct the attractor. Following Barkoulas et al. [50], given a discrete dynamic system of the form

(4) $$x_{t}=F\left(x_{t-1}\right),x\in {\mathbb{R}}^{n}\;$$

such that $F:U\to R^{n}$ is a function and *U* an open subset of ${\mathbb{R}}^{n}$ [52], a closed invariant set A⊂U  is an attracting limit set of U if there is an open neighborhood V of A, such that the limit set of iterates is *A*, ∀xϵV when t→∞.

Empirically, it is often observed as a series of scalar observations *y*, which represent the multidimensional system in Equation (4). In order to recover the dynamics of the system (i.e., original trajectory) by analyzing the observed time series *y_t_,* the Takens [53] embedding theorem is used, which is presented below.

An m-dimensional vector is defined and constructed from the observed time series.

(5) $$y_{t}^{m}=\left(y_{t},\ldots ,y_{t+m-1}\right)=\;\left(g\left(x_{t}\right),\ldots ,g\left(F^{m-1}\left(x_{t}\right)\right)\right)\equiv I_{m\;\;\;\;}$$

where $F^{m-1}\;$ is the composition of *F* with itself *m−*1 times. The idea is to reconstruct the state dimension space by expanding the one dimensional signal $y_{t}\;$ into an m-dimensional phase space, where each observation in the signal $y_{t}$ is replaced by the vector $y_{t}^{m}$ in Equation (5). 

Takens’ embedding theorem states that for each pair (F,g) the map $I_{m}:\;R^{n}\to R^{m}\;$ will be an embedding for m≥2n+1. This guarantees the existence of difeomorphisms between the original and the reconstructed attractor as long as the embedding dimension m is sufficiently large with respect to the dimension of the attractor. In short, Takens’ theorem assures that both attractors can be considered to represent the same dynamical system in different coordinate systems when m≥2n+1.

The main characteristics of the tools for detecting a chaotic regime used in this research are described below.

#### 3.2.1. Correlation Dimension

The Correlation Dimension arises as a response to the problem of estimating the dimensions to characterize a chaotic phenomenon. When reconstructing, it is necessary to know the number of dimensions, m, or the embedding dimension, both to make its representation in the phase diagram and to estimate a simple model of the phenomenon. An important characteristic of chaotic attractors is their dimension, which is defined as the lower limit of the number of state variables (degrees of freedom) needed to describe steady-state behavior. The correlation dimension test is a topologic one which measures a quantity called correlation dimension. It distinguishes chaotic series from random series by investigating the correlation dimension behavior of the data [30].

Let us consider the series ${\left\{y_{t}\right\}}_{t=1}^{n}$ and, from this, the sequence of N=n−m+1 m-dimensional vectors, $y_{t}^{m}=\left(y_{t},\ldots ,y_{t+m-1}\right)$ that gives the reconstructed series.

To estimate the dimension of the reconstructed attractor, the algorithm of Grassberger and Procaccia [47] is used, which is based on the correlation integral given by the following expression:

(6) $$C\left(\epsilon \right)=\frac{2}{N\left(N-1\right)}\sum_{t<s}H\left\{\epsilon -||X_{t}^{m}-X_{s}^{m}||\right\}$$

where ||.||, *m* and *H* represent respectively a norm operator, the embedding dimension and the Heaviside function. There are several norms that can be used to measure the distance between two different state vectors, such as the Euclidean norm or the maximum norm.

To determine the correlation dimension, it is needed to determine how $\;C_{\;}\left(\varepsilon \right)$ changes as ϵ changes. As ϵ grows, the value of $\;C_{\;}\left(\varepsilon \right)\;$ grows because the number of near points to be included increases. Grassberger and Procaccia [47] showed that for sufficiently small ε, $C_{\;}\left(\varepsilon \right)\;$ can be well approximated by $C_{\;}\left(\varepsilon \right)\sim \varepsilon^{v}$_)_. In other words, when ε→0, $C_{\;}\left(\varepsilon \right)\;$ grows at rate v where v is the value of the correlation dimension (CD). The estimate of v when m→∞*,* provides the correlation dimension (CD). The dimension of a dynamic system is determined by estimating the slope of the regression of $ln_{\;}C_{\;}\left(\varepsilon \right)$ versus log ϵ and an intercept for small values of ε  and depends on the chosen embedding dimension. If the data are purely stochastic, the correlation dimension will equal m for all m. If the data are deterministic, the estimated slope will stabilize at one point, not increasing as m increases. This “saturation” of the slope is the estimated correlation dimension for the unobserved process, which underlies the process that generated the data. That is, if the dynamic system is chaotic, $C_{\;}\left(\varepsilon \right)$ will stabilize at some point D, as m grows.

#### 3.2.2. Lyapunov

Lyapunov’s exponents measure the average rate of divergence or convergence between two adjacent orbits, i.e., they quantify the sensitivity to initial conditions in the phase space, identifying the basic attribute of deterministic chaos. It is approached through an exponential function, in which the exponent determines the rate of divergence of adjacent orbits that start from close points in an infinitesimal way. Chaotic systems exhibit a positive coefficient λ and systems that are stable, a negative coefficient. 

The Lyapunov exponent is one of the most employed techniques to assess the presence of chaotic behavior in time series [16]. Specifically, the largest Lyapunov exponent can be used to measure the rate of separation of closed trajectories and estimate the overall degree of chaos of a nonlinear dynamical system [54].

There are many algorithms in the literature for the estimation of this coefficient. The algorithm proposed by BenSaïda and Litimi [14] is followed in this research. Specifically, it has been empirically proven that the methodology adopted in this article to determine the Lyapunov exponent (λ) is the one that behaves best for noisy series [55]. Indeed, to estimate λ from experimental or observational data, there are two main classes of methods, both of which are based on reconstructing the space state by the delay coordinates methods. The direct methods are based on the calculation of the growth rate of the difference between two trajectories with an infinitesimal difference in their initial conditions [56]. Among the limitations of this method is that it cannot accept measurement errors or noise [57]. On the contrary, the Jacobian-based approach can give consistent estimates of the Lyapunov exponents even in the presence of noise [58,59]. The Jacobian method is based on nonparametric regression to estimate the Jacobians and λ. It consists of computing the Jacobian matrix of the chaotic map. However, for a scalar time series, the map generating the process is usually unknown; as a result, the Jacobian matrix could not be estimated, and the Lyapunov exponent cannot be computed. For that purpose, it is needed to approximate the unknown chaotic map with a known function. McCaffrey et al. [58] compared several alternatives: thin-plate splines, neural nets, radial basis functions and projection pursuit. Based on the simulations performed by them, neural net was the best regression method for chaotic systems with noise. In this line, Bailey et al. [59] proposed a regression method which involves the use of neural networks. Simulation results for a noisy Henon system suggest that the neural net regression method yields accurate estimates values of the Lyapunov exponent.

In order to shrink the noise in a dynamical system, a wavelet-based denoising method to filter the data has been employed by several researchers. In particular, the theory of signal denoising using wavelets has been developed by Donoho and Johnstone [60]. Garcin and Guégan [61] adapted the theory for signals in which the noise influence is nonlinear and the wavelet transform-based detection of chaos has been proposed by Rubežić et al. [62]. While this approach could be appropriate for physical systems where noise is an intruder of the real pure signal, for financial data, where noise is an inherent property to markets, denoising the data could modify some of the stylized financial facts that have been discussed earlier in the paper and alter the true dynamics that underlie the time series to be tested [63]. Hence, following this reasoning, a neural network approach has been chosen in this research.

Briefly, the methodology consists in the following steps: let us consider a time series ${\left\{y_{t}\right\}}_{t=1}^{T}\;$ represented as follows:

(7) $$y_{t}=f\left(y_{t-L},y_{t-2L},\ldots ,\;y_{t-mL}\right)+\varepsilon_{t}$$

where *L*, *m*, *ε* and *f* stand for the time delay, the embedding dimension, noise added to the series and an unknown chaotic map, respectively and *t* is the time script. The Lyapunov exponent (LE) is defined as:

(8) $$\hat{\lambda }=\frac{1}{2M}\ln\left(v_{1}\right)$$

where the “block length” M is the number of evaluation points used for estimating the Lyapunov exponent which stands for an arbitrarily selected number of observations and $v_{1}$ is the largest eigenvalue of the matrix ${\left(T_{M}U_{0}\right)}^{\prime }\left(T_{M}U_{0}\right),$ with $T_{M}=\overset{M-1}{\underset{t=1}{\prod }}J_{M-t}$ $J_{t}$, the Jacobian matrix of the chaotic map and $U_{0}=\;{\left(1,\;0,\;\ldots ,\;0\right)}^{\prime }$.

Because f is usually unknown, it is needed to approximate the Jacobian matrix. The authors employ a single-layer feed-forward neural network using nonlinear least squares for different values of *m* = 1…8 and later calculate the LE spectrum. Hence, the chaotic system is estimated by the following equation:

(9) $$y_{t}\approx \alpha_{0}+\overset{q}{\underset{j=1}{\sum }}\alpha_{j}\tan h\left(\beta_{o,j}+\overset{m}{\underset{i=1}{\sum }}\beta_{i,j}y_{t-iL}\right)+\varepsilon_{t}$$

(*L*, *m q*) are selected as the triplet that provides the highest value for λ and are associated with the complexity of the system. The test for chaos is then constructed based on the asymptotic distribution of *λ* [56].

#### 3.2.3. 0/1 Method

The 0/1 method for chaos was developed to distinguish between regular and chaotic dynamics in deterministic dynamical systems. This tool for chaos was proposed by Gottwald and Melbourne [46] and has a series of advantages over other tests, such as the fact that it is not necessary to reconstruct the phase space or that the result, which is 0–1, is very easy to interpret. Also, rather than requiring phase space reconstruction which is necessary to apply standard Lyapunov exponent methods to the analysis of discretely sampled data, the technique works directly with the time series and does not involve any preprocessing of the data. 

The input of the test is a one-dimensional time series y(t) for t = 1, 2,3, … The data y(t) is used to drive the following two-dimensional system:

(10) $$p\left(t\right)=\sum_{j=1}^{t}y\left(j\right)\cos(y\left(j\right))\;t=1,2,3,\ldots$$

(11) $$q\left(t\right)=\sum_{j=1}^{nt}y\left(j\right)\sin(y\left(j\right))\;t=1,2,3,\ldots \;$$

Define the (time-averaged) mean square displacement (MSD)

(12) $$M\left(t\right)=\lim\frac{1}{N}{\left[p\left(j+t\right)-p\left(j\right)\right]}^{2}\;t=1,2,3,\ldots$$

If the system is chaotic, then M(t) will grow linearly over time. If the system is not chaotic, M(t) will be bounded. The asymptotic growth rate of MSD is defined as:

(13) $$K=\underset{t\to \infty }{\lim}\frac{\log\;M\left(t\right)\;}{\log\;\left(t\right)\;}$$

*K* can be determined numerically by a linear regression of log (*M*(*t*)) versus log (*t*). Under general conditions, the limits *M*(*t*) and *K* can be shown to exist, and *K* takes either the value *K = *0 signifying regular dynamic or the value *K* = 1 signifying chaotic dynamic.

#### 3.2.4. MGRM Test

The MGRM Test is explained in detail in [6]. As the authors of the method argue, it is a test for the deterministic process as opposed to the stochastic one which is based on symbolic dynamics and entropy. The MGRM test is based on the concept of permutation entropy, which has its roots in symbolic dynamics. The basic idea behind symbolic dynamics consists of dividing the phase space into a finite number of regions and labeling each region with an alphabetical symbol. A relevant property of symbolic dynamics is that essential features of the underlying dynamics, such as its deterministic or stochastic nature or its complexity, are preserved. Likewise, entropy accounts for the unpredictability of the system under study, which is a crucial feature of complex systems. 

In the first instance the notation is introduced: let ${\left\{y_{t}\right\}}_{t\in T}$ be a stationary time series, ${\left\{y_{t}\right\}}_{t\in I}$ an observation with I={1,…,T}  and m the embedding dimension with m ≥2. Ordinal patterns will be defined for “m”. To that end, the scalar time series is embedded to an m-dimensional space: $Y_{m}\left(t\right)=\left(y_{t},y_{t+1},\ldots ,y_{t+\left(m-1\right)}\right)\;for\;t\in T.$ The ordinal pattern of embedding dimension m, at a given time *t* is defined as the unique permutation $\pi_{m}\left(t\right)\equiv \left(r_{0}\;r_{1}\ldots r_{m-1}\right)\;$ of the set {0, 1, …, m−1}  satisfying:

(14) $$y_{t+r_{0}}\leq y_{t+r_{1}}\leq \cdots \leq y_{t+r_{m-1}\;}$$

(15) $$r_{s-1}<r_{s}\;\;\mathrm{if}\;y_{t+r_{s-1}}=\;y_{t+r_{s}\;}$$

By means of (15) it is guaranteed the uniqueness of the permutation defined by (14). So, the vector or *m*-history $Y_{m}\left(t\right)\;$ is converted into a unique *symbol* $\pi_{m}\left(t\right)$. In fact, $\pi_{m}\left(t\right)\;$ describes how the order of the dates: t+0<t+1<…<t+(m−1) is turned into the order of the corresponding analyzed values. The basic idea is to divide naturally the state space in which the dynamics takes place into a finite number of partitions using the time-dependent information contained in the *m*-history $Y_{m}\left(t\right)\in R^{m}$. According to the previous definition, partitions depend on the ordinal structure of the *m*-history. In particular, $\pi_{m}\left(t\right)=\pi_{m}\left(s\right),\;s\neq t,\;$ if and only if for all k,I ϵ {0,1,…,m−1} with k≠I it holds that $y_{t+1}\leq y_{t+k}↔y_{s+1}\leq y_{s+k}.\;$

In general, given a time series ${\left\{y_{t}\right\}}_{t\in T},\;$ all *m*! permutations of order *m* are considered here as possible order types of *m* different numbers. Then the relative frequency or unconditional success probability p(π)  of each symbol or permutation π for a given time series and an embedding dimension parameter *m* exists and it can be defined as:

(16) $$p\left(\pi \right)=\frac{card\left\{t\;|\;0\;\leq \;t\;\leq \;T-\left(m-1\right),\;Y_{m}\left(t\right)\;has\;type\;\pi \;\right\}}{T-m+1}$$

Given an embedding dimension  m≥2, modified Shannon entropy that stands for the *m*! distinct symbols is defined as follows:

(17) $$h\left(m\right)=-\sum_{i=1}^{m!}p\left(\pi_{i}\right)\log p\left(\pi_{i}\right)\;\;$$

The test is constructed as follows: Let *m* be embedding dimension and *T* be the number of observations and fix w,k∈ℕ such that $w=\frac{m!}{k}$. Next, the subsets $W_{j}$ that must verify $W_{1}\subseteq W_{2}\subseteq \cdots \subseteq W_{n}\;$ in the following way $W_{j}=W_{j-1}\cup^{\;}\left\{\;w\;symbols\;chosen\;at\;random\;in\;S_{m}\;\backslash \;W_{j-1}\right\}$ for *j* = 2, …,*k*. are constructed.

Subsequently the next modified permutation entropy’s function is calculated as:

(18) $$h^{W_{j}}\left(m\right)=-\underset{\pi_{j}\in w_{j}}{\sum }p\left(\pi \right)\log p\left(\pi \right)$$

The authors of the method showed that by analyzing the modified permutation entropy’s function values it is possible to distinguish and identify deterministic systems. In this process no more information is gained by increasing the number of symbols under consideration. In contrast, in non-deterministic process, this information or complexity is increased.

This latter property is tested in the following way: Let $dh^{W_{j}}\left(m\right)$ be the $h^{W_{j}}\left(m\right)\;slope$:

(19) $$dh^{W_{j}}\left(m\right)=\frac{h^{W_{j+1}}\left(m\right)-h^{W_{j}}\left(m\right)}{\log\left(\frac{j+1}{j}\right)}$$

When considering random process, the numerical slope of permutation entropy will increase with *(log(jw)*), while this will not hold for chaotic or regular processes.

The property described above that identifies deterministic systems $h^{W_{j}}\left(m\right)$ is checked by carrying out the following regression:

(20) $$dh^{W_{j}}\left(m\right)=\alpha_{0}+\alpha_{1}j+\varepsilon_{j}\;for\;j=1,2,\ldots ,\;k-1\;$$

where $\;\varepsilon_{j}$ is white noise. As a result, the estimated parameter ${\hat{\alpha }}_{1}$ can be used to evaluate $dh^{W_{j}}\left(m\right)$ increases with *j*. In mathematic notation, the test is the following one:

(21) $$\begin{array}{c} H_{0}\equiv \alpha_{1}=0\\ H_{1}\equiv \alpha_{1}>0 \end{array}\;$$

Indeed, regression (20) can be considered as a simple symbol-trend model. As in the simple time-trend model, the ordinary least squares (OLS) estimate ${\hat{\alpha }}_{1}$ is so that asymptotically the usual *t*-test of *H*_0_: ${\hat{\alpha }}_{1}=0\;$ is valid. If the coefficient obtained is null, then the series is deterministic; otherwise (greater than 0), it is stochastic [6].

#### 3.2.5. Recurrence Plots

In addition to the analytical methods described above, there are other methods for detecting chaos and other patterns of a more visual nature, the so-called recurrence plot (RP) being one of the most widely used.

The Recurrence Plot (RP) is an analysis tool that reveals the existence of recurrent and intermittent patterns in time series. First proposed by Ekmann et al. [64], it has been widely applied in the characterization of dynamic systems. This topological method shows the hidden structures of time series from a qualitative point of view. The plots are constructed by assuming mutual distances that belong to the same path in the reconstructed phase space [17]. 

The construction is done as follows: 

Let *m* be the embedding dimension and $y_{t}^{m}$ the vector m-dimensional in the reconstructed phase space in time *t* = 1, 2, …, k

The recurrence matrix is generated by comparing each embedded vector $y_{i}^{m}\;$ with the other $y_{j}^{m}.$ A point is drawn if this comparison is less than a value ε for a specific distance. That is, if the condition is met: $||y_{i}^{m}-y_{j}^{m}||<\varepsilon$. 

Recurrence of a moment of state *i* at a different time *j* occurs when $y_{j}^{m}\;$ is close enough to $y_{i}^{m}$. The RP can therefore show which vectors are close together and which are far apart.

The diagonal structures of the RP identify the range in which a fraction of the path is relatively close to another at a different time. For a deterministic chaos system, small lines are observed parallel to the main diagonal. However, in random systems, small line segments are absent, and evenly distributed points are shown. 

This technique is independent of some constraints such as sample size, noise and stationarity [11]. It also provides additional information about the structure of the attractor, since the plot preserves the temporal order of the series, allowing the place and periodicity of the periodic orbits to be known.

## 4. Empirical Results

Applying the methodological framework described above, the main results obtained are shown below.

### 4.1. Data

**Figure 1:**
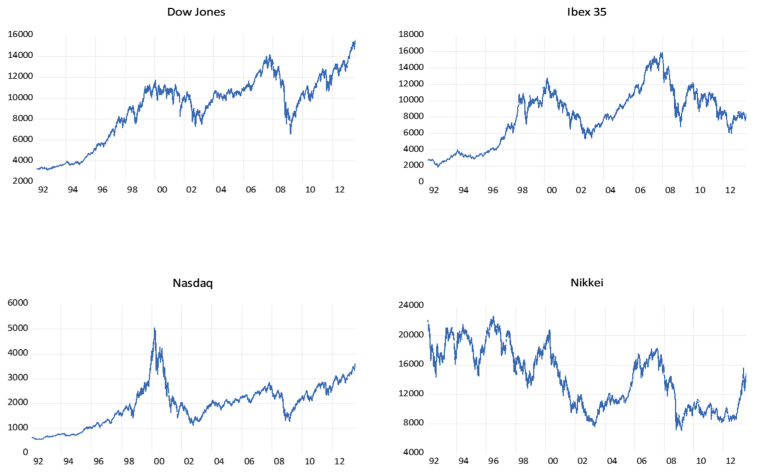
Plot of stock indices series. Time points (year) are on x-axis and observations are on y-axis. Source: Yahoo finance.

Four different daily time series are analyzed, using the closing prices of the following major stock market indices: the Dow Jones Industrial Average (Dow Jones), the Ibex 35 (Ibex), the Nasdaq 100 (Nasdaq) and the Nikkei 225 (Nikkei). All the data were obtained from Yahoo Finance and covers the period from 1 January 1992 until 31 July 2013. Figure 1 shows the evolution of the series over the sample period.

Table 3 presents the main descriptive statistics of the series studied. Both the series of the main stock market index of the U.S. Stock Exchange and that of the Spanish Stock Exchange show a negative asymmetry coefficient (−0.51 and −0.05, respectively), unlike the other financial series considered in the study. Moreover, it can be concluded that the data do not come from a normal distribution, as reflected by the Jarque–Bera test (*p*-value < 0.05).

### 4.2. Returns

**Figure 2:**
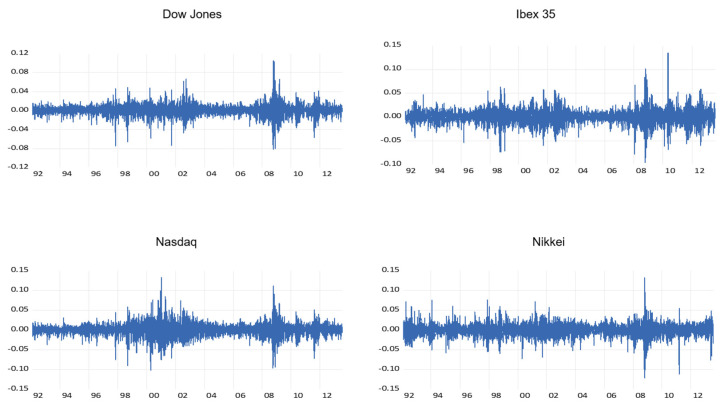
Plot of stock indices returns series. Time points (year) are on x-axis and observations are on y-axis. Source: Own work.

After testing that the series are not stationary (see Table 4), the returns from each series were obtained, by considering the logarithms of the ratio of two consecutives prices. Figure 2 shows the time development of the returns. They show an excess of kurtosis, a negative skewness coefficient (Table 3) and more fat-tails in comparison with those from a normal distribution. These characteristics are typical of financial returns [2]. Excessive kurtosis means that returns far from the average are more common than in a normal distribution and therefore the investor is subject to greater risk. The Jarque–Bera test indicates departure from the normal distributions for all the cases. According to the results from the Augmented Dickey–Fuller, Philips–Perron and KPSS tests, all the returns series are stationary (Table 4).

### 4.3. Linear Dependence

As some methodology (for example the BDS test) is not robust to the presence of linear relationships, the linear dependence was removed using ARMA models. Following the criterion described by Barkoulas et al. [51], the best ARMA model was selected for each series (Table 5). The selected fitted models for each of the series (ARMA (2,5); ARMA (0,3); ARMA (2,2) and ARMA (0,1), for the series Dow Jones, Ibex, Nasdaq and Nikkei respectively) are shown in Table 5 that shows all the models fitted in this research. Details of each model are presented in Supplementary Materials A (Tables SA1, SA4, SA7 and SA10). According to the results from the Augmented Dickey–Fuller, Philips–Perron and KPSS tests, applied on the residuals of the selected model and shown in Table 4, all the residuals series (ARMA) are stationary.

### 4.4. Nonlinear Dependence

Next, Keenan, Tsay, Teräsvirta, White, BDS and Kaplan methods were used to study nonlinearity. The principal advantage of using a broad set of instruments is to obtain the most information possible about the nature of the series, since to date none of the methods has proved successful in detecting all types of dependence. Nevertheless, some tests, such as the BDS, White or Kaplan, are more efficient in detecting dependency [51].

The BDS test was applied once the linear dependence was eliminated, as previously suggested by Brock et al. [51]. In this way the BDS test served as an indirect method of analyzing nonlinearity: if it rejected the null hypothesis, then the series was proven to be nonlinear. This procedure is theoretically feasible, since the calculations on the residuals of an autoregressive model do not lose the relevant information derived from the original series if the latter comes from a nonlinear chaotic system [51]. The BDS test is applied taking into account the different embedding dimensions (range: 2–9). The results are presented in Supplementary Materials B (Tables SB3–SB14).

Kaplan’s method is applied to a series in which the functional form that generated the series is unknown. The method’s aim is to determine whether there is evidence of an underlying deterministic mechanism, i.e., that the hypothesis that is tested is the stochastic linearity of the process. It is considered that the null hypothesis is verified if the value of the statistic K, calculated for the original series (K test), is smaller than the values obtained for the surrogates of each series, defined as the smallest value (Kmin) between the minimum and the mean, minus two standard deviations (KS). Several different values for both the delay and the embedding dimension parameters were taken into account, and the conclusions did not differ (see Supplementary Materials B).

Table 6 presents a summary of results for the nonlinear analysis. All the procedures used, except for Kaplan, suggest the existence of nonlinearity in the Dow Jones Index ARMA series. A similar pattern is observed for the Nasdaq Index. Further, as all the tests indicate some nonlinear dependence in the Ibex and Nikkei indexes, the data seems to present some type of dependence, since the BDS test shows significant results for the different embedding dimensions and the epsilons considered. For the latter, the White and Teräsvirta tests confirm the existence of dependence.

### 4.5. Volatility Clustering

The next step consists of modelling the conditional variance by fitting ARCH family models (GARCH and EGARCH). In first instance, the best GARCH (p,q) model was selected so as to adhere to the previously described criterion [51]. The selected models were GARCH (2,1) for the Dow Jones, Ibex and Nasdaq indexes and the GARCH (1,1) for the Nikkei Index (Table 5). After fitting each model, standardized residuals were obtained (from now on GARCH and EGARCH series). Unlike GARCH models, EGARCH models can be used to estimate the conditional variance, taking into consideration the sign of the innovation of the previous period. These types of models successfully capture the asymmetric response in the conditional variance and, hence, are suitable candidates for modeling financial processes. The chosen models were EGARCH (2,1), EGARCH (2,3), and EGARCH (1,1) for the Dow Jones and Ibex indexes, the Nasdaq Index and the Nikkei series, respectively (Table 5 and Supplementary Materials A). The standardized residuals of all the series are less leptokurtic (with an average value of 4) than those obtained from the ARMA models (Table 3).

Since nonlinearity is a necessary, but not sufficient, condition for chaotic behavior, its existence was first analyzed in residuals of the volatility models (Table 6 and Supplementary Materials B). In general, linear hypothesis is not rejected, but nonlinearity was found in some cases. Indeed, the BDS test shows some remaining dependence in the Dow Jones Index, which is compatible with the existence of chaos in the EGARCH model residuals. The Tsay and BDS tests reject linearity for the Ibex Index in the GARCH and EGARCH models, respectively. Likewise, some procedures show evidence of nonlinearity in the case of the GARCH and EGARCH models for the Nasdaq series. Finally, for the Nikkei Index, the BDS rejects the hypothesis of independence for all the cases considered.

### 4.6. Chaotic Behavior Analysis

The following methods were used to test the existence of chaotic dynamics. Results are presented in Supplementary Materials C.

Correlation Dimension

The correlation dimension (CD) [48] quantifies the degree of complexity of a system and distinguishes a deterministic system from a stochastic one. The results suggest that, in all cases except for the EGARCH model in the Dow Jones Index, the CD increases as the embedding dimension increases, however, the CD is below the expected value for a random process (Supplementary Materials Table SC1). Moreover, except in the latter series, the saturation of the slope as the embedding dimension increases is not observed. Thus, it is not possible to assure that the series are chaotic. Overall, there is sufficient evidence against the existence of a strange attractor; and if this is the case, it might be of a high dimension.

*Lyapunov Test*

The Lyapunov exponent reflects the average rate of convergence or divergence of two paths that are, initially, points that are very close in the phase space. Positive values indicate the existence of chaotic dynamics. Here, the algorithm described by BenSaïda and Litimi [14] was used. Negative significant Lyapunov exponents were obtained in all cases (Supplementary Materials C, Table SC2). Thus, the assumption of chaotic behavior was rejected in all cases.

0/1 method

Following the procedure described by the authors of the method, which consists of using several frequencies to increase the degree of robustness of the test, the test was carried out a total of 6000 times and the median of all realizations was taken [46]. The results of the 0/1 method are shown in Supplementary Materials Table SC3. The test provides a value close to 0 or 1. If the value is 0 it is concluded that the series is clearly stochastic, however if the result is 1, it cannot be stated with certainty that the series has a chaotic component, since the result may be due to noise. All the results obtained are close to value 1 (Supplementary Materials Table SC3), so it is concluded that the series may have a lot of noise or may have a significant chaotic component.

*MGRM Test*

This test is constructed as follows. First the Shannon modified entropy values are calculated. Then, the information or complexity is estimated by a linear regression that used the entropies as the explanatory variable. The coefficient of the entropies is then analyzed as in deterministic series the complexity derived from the entropy of permutation does not increase when the number of symbols increases, once the saturation point is reached. To conduct the test, the parameters were fixed such that m = 4, k = 2, and w = 12. The results were positive in all the cases (Supplementary Materials Table SC4). Thus, the hypothesis of determinism is rejected.

Recurrence plots

Visual Recurrence Analysis is based on Eckmann’s [48] definition of a recurrence graph. The degree of complexity and the existence of chaos is analyzed by generating the recurrence plots. This kind of analysis can capture the recurrence property of states, one of the essential properties observed in chaotic systems. The points above the main diagonal, representing the distance between the same vector and each embedded vector in the phase space and therefore segments parallel to the diagonal, would indicate a chaotic behavior [50].

**Figure 3:**
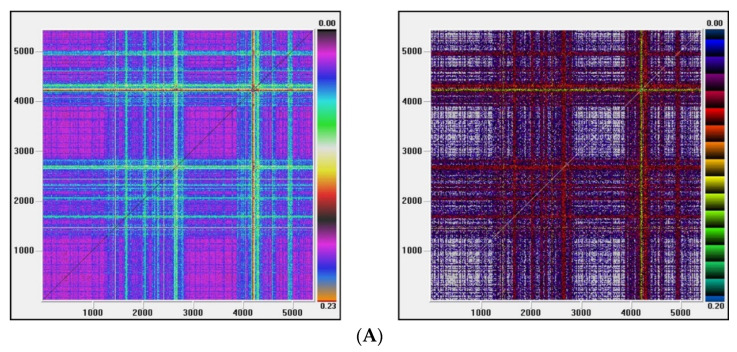

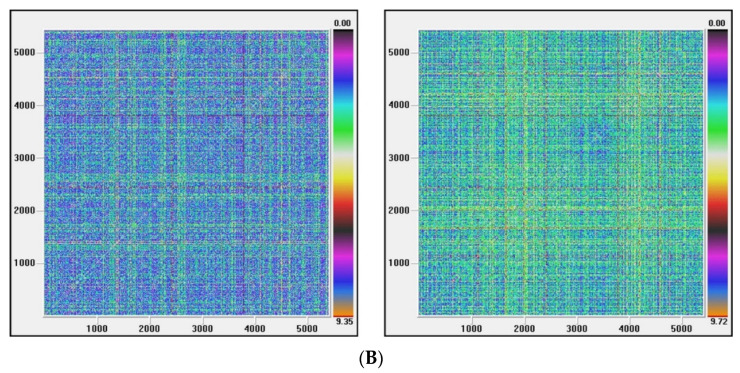
(**A**) Recurrence plot for the returns and ARMA series from the Dow Jones index; (**B**) Recurrence plot for the GARCH and EGARCH series from the Dow Jones index.

**Figure 4:**
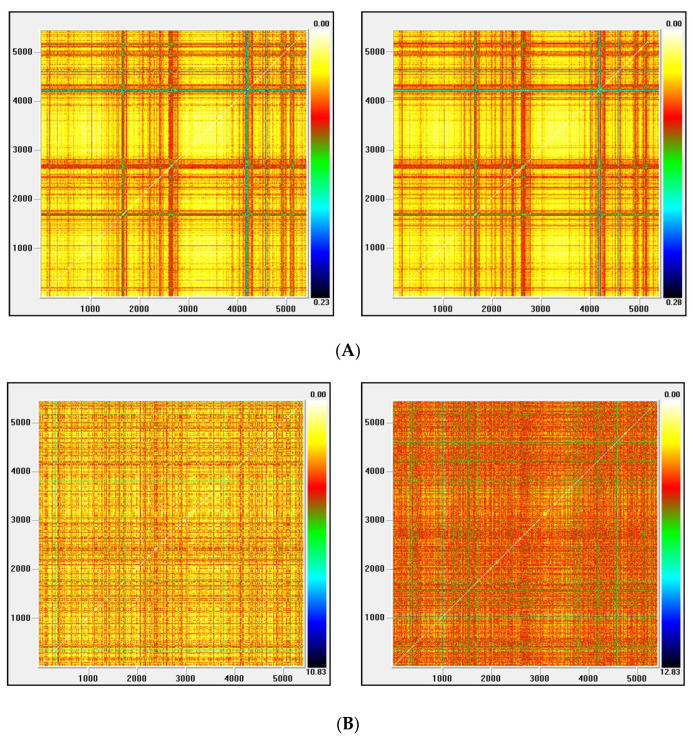
(**A**) Recurrence plot for the returns and ARMA series from the Ibex index; (**B**) Recurrence plot for the GARCH and EGARCH series from the Ibex index.

**Figure 5:**
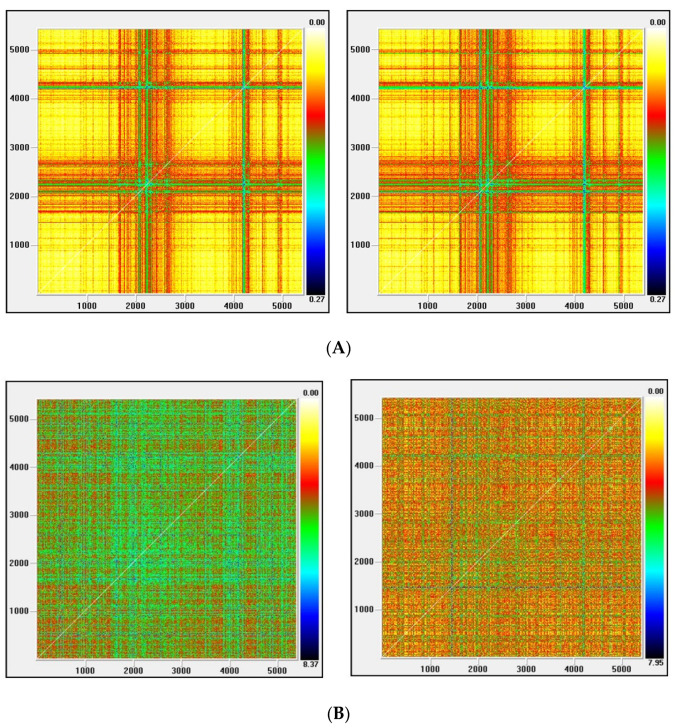
(**A**) Recurrence plot for the returns and ARMA series from the Nasdaq index; (**B**) Recurrence plot for the GARCH and EGARCH series from the Nasdaq Index.

**Figure 6:**
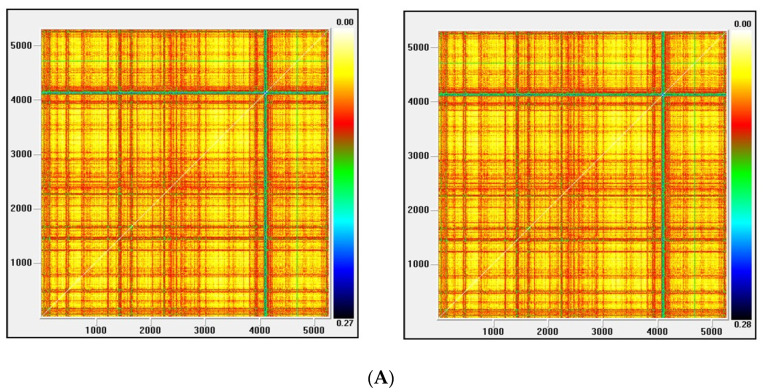

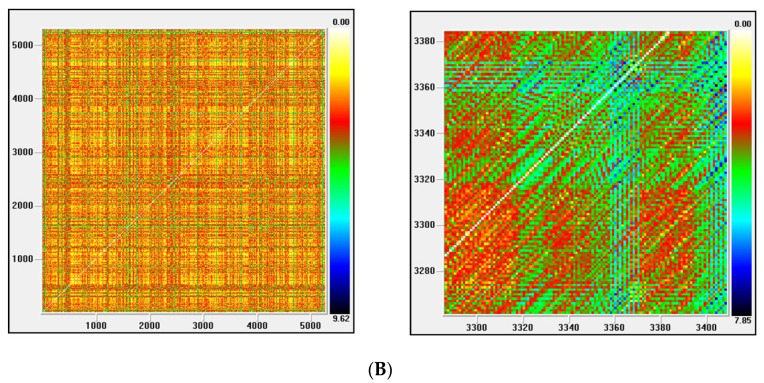
(**A**) Recurrence plot for the returns and ARMA series from the Nikkei index; (**B**) Recurrence plot for the GARCH and EGARCH series from the Nikkei Index.

Figure 3, Figure 4, Figure 5 and Figure 6 show the recurrence plots for the four series considered. For their elaboration, the optimal values of the two parameters, m—the embedding dimension and *τ*—the considered delay, as previously described, have been considered. Likewise, the Euclidean distance has been chosen and the radius cut-off point (ε) is defined as 10% of the maximum distance between all the points of the reconstructed phase space. This value is adopted in several works, for example in Barkoulas et al. [50].

The recurrence plots can present different patterns, among them the presence of short segments parallel to the main diagonal are related with the existence of chaos. Except the one corresponding to the EGARCH model of the Nikkei stock, none of these patterns is observed in the series evaluated, concluding that the underlying system that generated the series apparently does not have a significant chaotic component.

## 5. Conclusions

This study examines the underlying dynamics of four of the principal stock exchange indexes and the asset markets that they represent. To determine the existence of a nonlinear and chaotic regime in the analyzed time series, a comprehensive methodological framework has been adopted that integrates a great number of tools. Thus, seven techniques have been used for nonlinear analysis and five for the analysis of chaotic behavior, including those most suitable for noisy time series. Results support the existence of nonlinearity, which is not consistent with chaos. In addition, GARCH/EGARCH models explain a significant part of the nonlinear structure that is found in the four stock markets analyzed.

The findings are in concordance with the conclusions of other researchers who use suitable procedures for noisy series to detect chaos in stock markets (e.g., [14]). On the other hand, our conclusion contradicts the findings of previous studies that found evidence of low dimensional chaos but used several techniques that do not account for noise (e.g., [66]).

The analysis of the stock exchange indexes carried out in this research is of great interest because the markets they represent are currently a faithful barometer of the evolution of activity in the most developed economies. Likewise, the modelling and study of the existence of nonlinearity and the chaotic dynamics of stock markets is particularly useful for agents involved in capital markets: investors, financial intermediaries, credit institutions, regulators, etc. Knowledge of their dynamics is essential for making accurate predictions of their future evolution and of crucial importance for properly managing the level of risk in the capital market and correctly and efficiently applying prices in the derivatives and futures markets.

Further research is needed to examine other types of both parametric and nonparametric nonlinear models, as well the relationships between the different stock market indices, using a multivariate GARCH model (MGARCH). Also, a new research would consist of the study of the effects of the collapse of stock prices caused by the COVID-19 outbreak on the dynamics of the series analyzed and see how this phenomenon would have affected the results obtained. Other further work could be to carry out the study dividing the total sample into several periods.

## Figures and Tables

**Table 1:** Results of research on nonlinearity and chaos in the stock market.

Authors (Year)	Series Considered	Tools Used	Results
Scheinkman and LeBaron (1989) [12]	They study several weekly and daily series of the US stock market.	BDS statistics. Correlation Dimension.	Evidence of nonlinearity. Evidence of chaos.
Hsieh (1991) [19]	He studies the weekly series of returns for a stock market portfolio.	BDS test. Correlation Dimension.	No evidence of IID. No evidence of chaos.
Blank (1991) [20]	He analyzes the behavior of the S&P 500 stock index	Correlation Dimension. Lyapunov exponents test.	Evidence of chaos (presence of deterministic nonlinearity). This is a necessary but not sufficient condition for chaos, as there are no tests of statistical significance of the estimates.
Mayfield and Mizrach (1992) [21]	They analyze the time series of the high frequency returns in the S&P 500 index.	Correlation Dimension. Lyapunov test.	Evidence of chaos.
Yang and Brorsen (1993) [22]	They study several futures markets, including the S&P 500 stock index.	BDS. Brock’s residual test.	Evidence of nonlinearity. Evidence of chaos in about half of the cases studied.
Abhyankar et al. (1995) [23]	They study the presence of nonlinearity and chaos in the FTSE-100 index yield series.	Hinich test. BDS test. Lyapunov exponent test.	Evidence of nonlinearity. No evidence of chaos.
Sewell et al. (1996) [24]	They investigate the weekly changes in six major stock indices (the US, Korea, Taiwan, Japan, Singapore and Hong Kong) and the World Index.	Spectral analysis. Nonlinear dynamics techniques.	Evidence of nonlinearity in some of the time series. No evidence of chaos.
Abhyankar et al. (1997) [25]	They examine nonlinear dependence and chaos in the returns of the stock market indices: FTSE 100, S&P 500, NIKKE 225 and DAX.	BDS. Lee, White and Granger neural-network-based tests. Lyapunov exponent.	Evidence of nonlinearity. No evidence of chaos.
Barkoulas and Travlos (1998) [3]	They study the presence of a chaotic behavior in the Athens stock market.	BDS test. Correlation Dimension. Kolmogorov Entropy.	Evidence of nonlinearity. No evidence of chaos.
Pandey et al. (1998) [26]	They investigate the presence of chaos in five major European stock markets and the United States.	BDS. Rescaled Range (R/S) analysis.	Evidence of nonlinearity. No IID. No evidence of chaos.
Gao and Wang (1999) [27]	They study among others the daily futures series of the S&P 500.	BDS statistic. TAR-F Statistic. Q2 Test.	Evidence of nonlinearity. No evidence of chaos.
McKenzie (2001) [15]	He tests for the presence of chaos in 12 national stock market indices.	BDS test. Close returns test.	Evidence of nonlinearity. No evidence of chaos.
Kyrtsou and Terraza (2002) [28]	They examine the dynamics of the French stock market (CAC 40 Index).	Fractional integration test. Correlation dimension. Lyapunov exponents.	Evidence of nonlinearity. Evidence of chaos.
Antoniou and Vorlow (2005) [29]	They examine the noise-free versions of a set of FTSE 100 stock returns time series.	BDS statistic. Surrogate data analysis.	Evidence of nonlinearity. Inconclusive evidence of chaos.
Yousefpoor et al. (2008) [30]	They study the possible chaotic behavior of some selective stocks from the Tehran stock market.	BDS test. Lyapunov exponent test. Close returns test.	Evidence of nonlinearity. No evidence of chaos.
Matilla-Garcia and Ruiz Marin (2010) [6]	They study the chaotic behavior of the daily series of the Dow Jones Industrial Average stock market index.	0–1 test based on permutation entropy.	No evidence of chaos.
Mishra et al. (2011) [31]	This study tests for the presence of nonlinear dependence and deterministic chaos in the rate of returns series for six Indian stock market indices.	Test of independence. Variance ratio test. Hurst exponent. BDS test. Lyapunov exponent test.	Evidence of nonlinearity. Evidence of chaos in two out of six cases.
BenSaïda (2012) [32]	He investigates the existence of chaotic dynamics in the S&P 500, Nikkei 225 and CAC 40 stock index during the period 1999–2008.	A new methodology to apply Lyapunov’s exponent method.	No evidence of chaos.
Webel (2012) [33]	He analyzes chaotic behavior daily log returns of the 30 DAX members.	0–1 test.	Evidence of chaos.
BenSaïda and Litimi (2013) [14]	They examine nonlinearity and chaotic behavior in the following indices S&P 500, NASDAQ composite, Nikkei 225, CAC 40, FTSE 100 and DAX.	Lyapunov exponent (Neural network architecture is used in this test).	Evidence of nonlinearity. No evidence of chaos.
BenSaïda (2014) [34]	He investigates the existence of chaotic dynamics in the Standard and Poor’s 500 index returns over 4 different frequencies: weekly, daily, 30 min and 5 min basis.	Lyapunov exponent test.	No evidence of chaos.
Tiwari and Gupta (2019) [35]	They test for chaos in the historical daily and monthly datasets spanning over one century of stock returns for G7 countries.	0–1 test. Lyapunov exponent.	Evidence of chaos.

IId stands for independent and identically distributed. Source: Own work.

**Table 2:** Tools used.

Tool	Reference	Feature Tested
Augmented Dickey Fuller (ADF)	[36]	Stationarity (unit roots)
Phillips–Perron (PP)	[37]	Stationarity (unit roots)
Kwiatkowski–Phillips–Schmidt–Shin (KPSS)	[38]	Stationarity (unit roots)
Runs	[39]	Randomness
Keenan	[40]	Nonlinearity
Tsay	[41]	Nonlinearity
Teräsvirta	[42]	Nonlinearity
White	[43]	Nonlinearity
BDS	[18]	Independence; Randomness
	[44]	IID; Nonlinearity
Kaplan	[45]	Nonlinearity
0/1	[46]	Chaos
Correlation Dimension	[47]	Chaos
Lyapunov Exponent	[14]	Chaos
MGRM	[6]	Chaos
Recurrence Plots	[48]	Chaos

**Table 3:** Descriptive statistics.

	Original Series	Returns	ARMA	GARCH	EGARCH
DOW JONES					
Mean	9108.07	0.0003	−5.8 × 10^−7^	−0.0356	0.0028
Standard Deviation	3147.61	0.0112	0.0112	0.9994	0.9999
Median	10,080.3	0.0005	0.0004	0.0017	0.0350
Minimum	3136.58	−0.0821	−0.0790	−6.5989	−6.0801
Maximum	15,567.7	0.1051	0.0993	3.4852	3.7538
Skewness Coefficient	−0.5103	−0.1364	−0.2810	−0.4213	−0.3715
Kurtosis Coefficient	2.2721	11.4338	10.7387	4.4806	4.2585
Jarque–Bera Test	354.59 *	16,062.4 *	13,575.8 *	654.4 *	481.64 *
N	5415	5414	5412	5412	5412
IBEX					
Mean	8124.38	0.0002	4.86 × 10^−8^	−0.0407	−0.0063
Standard Deviation	3354.74	0.0146	0.0145	0.9989	1.0001
Median	8362.9	0.0007	0.0005	−0.0158	0.0166
Minimum	1873.58	−0.0959	−0.0965	−6.5618	−7.7301
Maximum	15,945.7	−0.1348	0.1335	5.9752	5.2568
Skewness Coefficient	−0.0526	−0.0054	−0.0428	−0.2057	−0.1960
Kurtosis Coefficient	2.3074	7.8791	7.7376	4.4174	4.4775
Jarque–Bera Test	110.96 *	5382.03 *	5076.16 *	492.48 *	528.23 *
N	5427	5426	5426	5426	5426
**NASDAQ**					
Mean	1950.235	−7.1910^−5^	−2.30 × 10^−7^	−0.0316	0.0043
Standard Deviation	842.569	0.0149	0.0156	0.0351	0.9999
Median	1989.22	−9.3410^-5^	0.0009	−6.1386	0.0706
Minimum	547.84	−0.1195	−0.1018	4.1826	−6.1143
Maximum	5048.62	0.1366	0.1265	0.9994	3.5622
Skewness Coefficient	0.3527	−0.0584	−0.0979	−0.3825	−0.4038
Kurtosis Coefficient	3.0323	12.7954	8.4806	4.0381	4.0070
Jarque–Bera Test	112.519 *	22,759.76 *	6782.03 *	374.96 *	375.74 *
N	5415	5414	5412	5412	5412
**NIKKEI**					
Mean	14,304.3	−9.122 × 10^−5^	−5.22 × 10^−8^	−0.0357	−0.0045
Standard Deviation	4073.77	0.0154	0.0154	0.9997	1.0001
Median	14,416.6	6.3110^-5^	0.0002	−0.0160	0.0129
Minimum	7054.98	−0.1211	−0.1204	−6.4953	−6.7925
Maximum	22,667	−0.1323	0.1282	5.6944	5.7235
Skewness Coefficient	0.1026	−0.2147	−0.2397	−0.1943	−0.1597
Kurtosis Coefficient	1.7032	8.2470	8.2517	4.4910	4.5000
Jarque–Bera Test	379.75 *	6104.3 *	6125.1 *	522.85 *	518.01 *
N	5287	5286	5286	5286	5286

Notes: Statistics for the time series considered in this study: original series, return series, and residuals of autoregressive moving average (ARMA), generalized autoregressive conditional heteroskedasticity (GARCH) and exponential GARCH (EGARCH) models. Skewness and kurtosis coefficient corresponds to the Fisher asymmetry coefficient and the kurtosis coefficient, respectively. *: The asterisk denotes significance at 5% level. Source: Own work.

**Table 4:** Stationarity analysis.

	Index	Returns
DOW JONES		
1. Augmented Dickey Fuller Test		
Constant	−1.0288 (0.7451)	**−56.0490 (0.0001)**
Constant and Linear Trend	−2.2115 (0.4825)	**−56.0525 (0.0000)**
2. Phillips–Perron Test		
Constant	−0.9623 (0.7685)	**−78.6723 (0.0001)**
Constant and Linear Trend	−2.1111 (0.5389)	**−78.6832 (0.0001)**
3. Kwiatkowski–Phillips–Schmidt–Shin Test		
Constant	7.1098	**0.1793**
Constant and Linear Trend	1.1008	**0.0832**
IBEX		
1. Augmented Dickey Fuller Test		
Constant	−1.8643 (0.3496)	**−53.5682 (0.0001)**
Constant and Linear Trend	−1.6676 (0.7655)	**−53.5897 (0.0000)**
2. Phillips–Perron Test		
Constant	−1.8167 (0.3727)	**−71.1395 (0.0001)**
Constant and Linear Trend	−1.5767 (0.8023)	**−71.1609 (0.0000)**
3. Kwiatkowski–Phillips–Schmidt–Shin Test		
Constant	5.1229	**0.2791**
Constant and Linear Trend	1.0088	**0.0543**
NASDAQ		
1. Augmented Dickey Fuller Test		
Constant	−1.3869 (0.5904)	**−54.9507 (0.0001)**
Constant and Linear Trend	−2.0781 (0.5574)	**−54.9485 (0.0000)**
2. Phillips–Perron Test		
Constant	−1.3869 (0.5904)	**−74.6870 (0.0001)**
Constant and Linear Trend	−2.0781 (0.5574)	**−74.6836 (0.0001)**
3. Kwiatkowski–Phillips–Schmidt–Shin Test		
Constant	0.0860	**0.1198**
Constant and Linear Trend	0.6694	**0.0861**
NIKKEI		
1. Augmented Dickey Fuller Test		
Constant	−2.3032 (0.1710)	**−75.6134 (0.0001)**
Constant and Linear Trend	−2.5121 (0.3222)	**−75.6142 (0.0001)**
2. Phillips–Perron Test		
Constant	−2.3485 (0.1568)	**−75.7549 (0.0001)**
Constant and Linear Trend	−2.5440 (0.3066)	**−75.7590 (0.0001)**
3. Kwiatkowski–Phillips–Schmidt–Shin Test		
Constant	5.5059	**0.0950**
Constant and Linear Trend	0.4156	**0.0491**

Augmented Dickey–Fuller *p*-values correspond to MacKinnon [65] one-sided *p*-values. For the Phillips–Perron test, lags were based on bandwidth Newey–West using Bartlett kernel. Critical values for the Kwiatkowski, Phillips, Schmidt and Shin test are 0.463 and 0.146 respectively for the constant and linear plus linear trend model. Significant values at the 95% confidence level are in bold.

**Table 5:** Models.

Index	Linear Model	Nonlinear Model	
	[ARMA]	[GARCH]	[EGARCH]
Dow Jones	ARMA (2,5)	GARCH (2,1)	EGARCH (2,1)
Ibex	ARMA (0,3)	GARCH (2,1)	EGARCH (2,1)
Nasdaq	ARMA (2,2)	GARCH (2,1)	EGARCH (2,3)
Nikkei	ARMA (0,1)	GARCH (1,1)	EGARCH (1,1)

**Table 6:** Summary of results for the nonlinear analysis.

Dow Jones					
Test	Ho	Returns	ARMA (2,5)	GARCH (2,1)	EGARCH (2,1)
Runs	Randomness	R	NR	R	R
Keenan	La	NR	R	NR	NR
Tsay	La	R	R	NR	NR
White	La	R	R	NR	NR
Teräsvirta	La	R	R	R	R
BDS	Lb	-	R	NR	Mixture
Kaplan	L	NR	Mixture	Mixture	NR
Ibex					
Test	Ho	Returns	ARMA (0,3)	GARCH (2,1)	EGARCH (2,1)
Runs	Randomness	NR	NR	NR	NR
Keenan	La	NR	NR	NR	NR
Tsay	La	R	NR	R	NR
White	La	NR	NR	NR	NR
Teräsvirta	La	R	R	NR	NR
BDS	Lb	-	R	NR	Mixture
Kaplan	L	Mixture	Mixture	NR	Mixture
Nasdaq					
Test	Ho	Returns	ARMA (2,2)	GARCH (2,1)	EGARCH (2,3)
Runs	Randomness	R	R	R	R
Keenan	La	R	NR	NR	NR
Tsay	La	R	R	R	R
White	La	R	R	R	R
Teräsvirta	La	R	R	R	R
BDS	Lb	R	R	Mixture	Mixture
Kaplan	L	R	Mixture	NR	NR
Nikkei					
Test	Ho	Returns	ARMA (0,1)	GARCH (1,1)	EGARCH (1,1)
Runs	Randomness	R	NR	R	R
Keenan	La	NR	NR	R	NR
Tsay	La	R	R	NR	NR
White	La	R	R	NR	NR
Teräsvirta	La	R	R	NR	NR
BDS	Lb	-	R	Mixture	Mixture
Kaplan	L	NR	NR	Mixture	Mixture

Ho: Null hypothesis. La: Linear in mean. Lb: This test can be applied as a linear test once the linear dependence has been eliminated. R represents that the null hypothesis is rejected; NR represents that the null hypothesis is not rejected. Mixture represents that the test reported no clear results. For the Kaplan method a total of 30 surrogates were generated from the original series, considering the same embedding dimensions and delay parameters.

## Supplementary Materials

The following are available online at https://www.mdpi.com/1099-4300/22/12/1435/s1, A: Details of the Models Applied to the Series; B: Results of the Randomness and NonLinearity tests and C: Chaotic Component Study.
