# Supplementary material for: A Comprehensive Framework for Uncovering Non-Linearity and Chaos in Financial Markets: Empirical Evidence for Four Major Stock Market Indices
Source: Entropy (Basel). 2020 Dec 18;22(12):1435. doi: 10.3390/e22121435 (PMC7767038; doi:10.3390/e22121435)
Supplement: Supplementary file 1 [file entropy-22-01435-s001.pdf]

ANNEX

A: Details of the Models Applied to the Series

A.1. Dow Jones

A.1.1. Linear Model

Table S.A1. ARMA (2,5) Model for the Dow Jones series

	Coefficient	Standard Deviation	T Statistic	P-value
C	0.0003	0.0001	2.2213	0.0264
AR(1)	-0.4288	0.2209	-1.9407	0.0523
AR(2)	0.3967	0.2115	1.8757	0.0608
MA(1)	0.3684	0.2206	1.6700	0.0950
MA(2)	-0.4673	0.1991	-2.3465	0.0190
MA(3)	0.0270	0.0271	0.9959	0.3194
MA(4)	0.0144	0.0174	0.8314	0.4058
MA(5)	-0.0620	0.0143	-4.3483	0.0000

A.1.2. GARCH Model

Table S.A2. GARCH (2,1) model for the Dow Jones series

	Coefficient	Standard Deviation	T Statistic	P-value
C	1.67E-06	1.88E-07	8.8708	0.0000
RESID(-1)^2	0.0400	0.0108	3.7079	0.0002
RESID(-2)^2	0.0653	0.0121	5.4057	0.0000
GARCH(-1)	0.8812	0.0069	127.578	0.0000

The linear model has been presented in the previous section.

A.1.3. EGARCH Model

Table S.A3. EGARCH (2,1) model for the Dow Jones series

	Coefficient	Standard Deviation	T Statistic	P-value
C(9)	-0.3417	0.0219	-15.5086	0.0000
C(10)	0.0173	0.0219	0.7923	0.4282
C(11)	0.1385	0.0227	6.0684	0.0000
C(12)	-0.1065	0.0059	-17.6231	0.0000
C(13)	0.9762	0.0019	516.3466	0.0000

The linear model has been presented in the previous section.

A.2. Ibex

A.2.1. Linear Model

Table S.A4. ARMA (0,3) model for the Ibex series

	Coefficient	Standard Deviation	T Statistic	P-value
C	0.0001	0.0002	1.1184	0.2634
MA(1)	0.0350	0.0136	2.5897	0.0096
MA(2)	-0.0453	0.0136	-3.3390	0.0008
MA(3)	-0.0447	0.0136	-3.2968	0.0010

SA.2.2. GARCH Model

Table S.A5. GARCH (2,1) model for the Ibex series

	Coefficient	Standard Deviation	T Statistic	P-value
C	3.04E-06	3.78E-07	8.0260	0.0000
RESID(-1)^2	0.0571	0.0137	4.1693	0.0000
RESID(-2)^2	0.0507	0.0153	3.3283	0.0009
GARCH(-1)	0.8796	0.0082	107.6930	0.0000

The linear model has been presented in the previous section.

SA.2.3. EGARCH Model

Table S.A6. EGARCH (2,1) model for the Ibex series

	Coefficient	Standard Deviation	T Statistic	P-value
C(5)	-0.3283	0.0250	-13.1625	0.0000
C(6)	0.0529	0.0257	2.0665	0.0388
C(7)	0.1076	0.0261	4.0997	0.0000
C(8)	-0.0813	0.0056	-14.5878	0.0000
C(9)	0.9769	0.0023	433.1963	0.0000

The linear model has been presented in the previous section.

A.3. Nasdaq

SA.3.1. Linear model

Table S.A7. ARMA(2,2) model for Nasdaq series

	Coefficient	Standard Error	T Statistic	P-value
C	0.0003	0.0001	1.5242	0.1275
AR(1)	-0.7502	0.0215	-34.838	0.0000
AR(2)	-0.9471	0.0212	-44.5772	0.0000
MA(1)	0.7512	0.0256	29.3533	0.0000
MA(2)	0.9240	0.0253	36.4630	0.0000

SA.3.2. GARCH Model

Table S.A8. GARCH (2,1) model for Nasdaq series

	Coefficient	Standard Deviation	T Statistic	P-value
C	2.3E-06	3.05E-07	7.6407	0.0000
RESID(-1)^2	0.0356	0.0121	2.9391	0.0033
RESID(-2)^2	0.0711	0.0137	5.1976	0.0000
GARCH(-1)	0.8832	0.0074	119.65	0.0000

The linear model has been presented in the previous section.

SA.3.3. EGARCH Model

Table S.A9. EGARCH (2,1) model for Nasdaq series

	Coefficient	Standard Deviation	T Statistic	P-value
C(6)	-0.3937	0.0389	-10.1108	0.0000
C(7)	0.0822	0.0247	3.3324	0.0009
C(8)	0.1612	0.0343	4.6953	0.0000
C(9)	-0.1173	0.0092	-12.7985	0.0000
C(10)	0.7692	0.1086	7.0815	0.0000
C(11)	-0.1781	0.1550	-1.1489	0.2506
C(12)	0.3857	0.0864	4.4660	0.0000

The linear model has been presented in the previous section.

A.4. Nikkei

A.4.1. Linear Model

Table S.A10. ARMA (0,1) model for the Nikkei series

	Coefficient	Standard Deviation	T Statistic	P-value
C	-9.11E-05	0.0002	-0.4496	0.6531
MA(1)	-0.0417	0.0137	-3.0304	0.0025

A.4.2. GARCH Model

Table S.A11. GARCH (1,1) model for the Nikkei series

	Coefficient	Standard Deviation	T Statistic	P-value
C	5.05E-06	5.66E-07	8.9170	0.0000
RESID(-1)^2	0.0960	0.0059	16.388	0.0000
GARCH(-1)	0.8838	0.0072	121.9457	0.0000

The linear model has been presented in the previous section.

A.4.3. EGARCH model

Table S.A12. GARCH (1,1) model for the Nikkei series

	Coefficient	Standard Deviation	T Statistic	P-value
C(3)	-0.3908	0.0303	-12.9040	0.0000
C(4)	0.1787	0.0100	17.7946	0.0000
C(5)	-0.0793	0.0046	-17.3603	0.0000
C(6)	0.9705	0.0030	320.6939	0.0000

The linear model has been presented in the previous section

B: Results of the Randomness and NonLinearity tests

B.1. Results for Runs, Keenan, Tsay, Teräsvirta and White tests

Table S.B1. Randomness and Non-Linearity tests (Dow Jones and Ibex)

**Dow Jones**				
**Test**	**Returns**	**ARMA (2,5)**	**GARCH (2,1)**	**EGARCH (2,1)**
Runs	**2818**	2689	**2812**	**2799**
	**0.0028**	0.6246	**0.0043**	**0.0124**
Keenan	0.5312[34]	0.0050[0]	0.5769[0]	0.6527[0]
	0.4661	0.9434	0.4476	0.4192
Tsay	**3.726**[34]	0.0433[0]	0.1724[0]	0.1079[0]
	**2.5E-140**	0.9880	0.9151	0.9555
Teräsvirta	**33.3379**	**30.0403**	**7.5198**	**11.0808**
	**5.7e-08**	**3e-07**	**0.0233**	**0.0040**
White	**29.8844**	**34.2144**	1.6772	4.5333
	**3.2E-07**	**3.7 E-08**	0.4323	0.1037
**Ibex**				
**Test**	**Returns**	**ARMA (0,3)**	**GARCH (2,1)**	**EGARCH (2,1)**
Runs	2674	2740	2778	2780
	0.2774	0.4802	0.0822	0.0731
Keenan	0.7947[16]	0.0054[0]	0.0045[0]	0.0052[0]
	0.3728	0.9412	0.9465	0.9430
Tsay	**3.296**[16]	0.0625[0]	0.1916[0]	0.1622[0]
	**4.5E-33**	0.9796	0.9022	0.9218
Teräsvirta	**18.3062**	**18.8244**	1.0816	3.7604
	**0.0001**	**8.2E-05**	0.5823	0.1526
White	2.5322	2.3671	0.7993	3.1827
	0.2819	0.3062	0.6705	0.2037

The first row shows the statistic associated with the test and in brackets. if necessary. the dimension of the model chosen to carry out the corresponding test. The second row shows the p-value.

Table S.B2. Randomness and Non-Linearity tests (Nasdaq and Nikkei)

**Nasdaq**				
**Test**	**Returns**	**ARMA (2,2)**	**GARCH (2,1)**	**EGARCH (2,3)**
Runs	**2573**	**2574**	2652	**2600**
	**0.0002**	**0.0003**	0.1348	**0.0036**
Keenan	**6.0446[37]**	0.2973[0]	**10.055[0]**	**6.695[0]**
	**0.0140**	0.5856	**0.0015**	**0.0097**
Tsay	**3.751[37]**	0.0487[0]	**4.287[0]**	**4.292 [0]**
	**3.4E-159**	0.9858	**0.0050**	**0.0050**
Teräsvirta	**55.2206**	**54.4824**	**11.978**	**7.3827**
	**1.0E-12**	**1.48E-12**	**0.0025**	**0.0250**
White	**25.2802**	**48.2101**	**12.593**	**8.3566**
	**3.239E-06**	**3.40E-11**	**0.0018**	**0.0153**
**Nikkei**				
**Test**	**Returns**	**ARMA (0,1)**	**GARCH (1,1)**	**EGARCH (1,1)**
Runs	**2785**	2695	**2741**	**2741**
	**0.0001**	0.1606	**0.0076**	**0.0076**
Keenan	2.7881[2]	0.0103[0]	1.8218 [0]	1.2816[0]
	0.0950	0.9192	0.1772	0.2577
Tsay	**7.94[2]**	0.0354[0]	0.2785[0]	0.2562[0]
	**2.8E-5**	0.9911	0.841	0.8569
Teräsvirta	**22.5858**	**22.0179**	1.1980	3.3999
	**1.2E-5**	**1.66E-05**	0.5494	0.1827
White	**19.7349**	**16.3223**	1.7512	3.3411
	**5.184E-05**	**0.0003**	0.4166	0.1881

The first row shows the statistic associated with the test and in brackets. if necessary, the dimension of the model chosen to carry out the corresponding test. The second row shows the p-value.

B.2. Results of BDS test

S B.2.1. Dow Jones series

Table S.B3. BDS test results for ARMA (2,5) series

**Épsilon/M**	**0.5*σ**	**1*σ**	**1.5*σ**	**2*σ**
2	**0.0077**	**0.0161**	**0.0165**	**0.0129**
	**0.0000**	**0.0000**	**0.0000**	**0.0000**
3	**0.0100**	**0.0339**	**0.0409**	**0.0339**
	**0.0000**	**0.0000**	**0.0000**	**0.0000**
4	**0.0079**	**0.0438**	**0.0634**	**0.0566**
	**0.0000**	**0.0000**	**0.0000**	**0.0000**
5	**0.0053**	**0.0476**	**0.0823**	**0.0791**
	**0.0000**	**0.0000**	**0.0000**	**0.0000**
6	**0.0033**	**0.0470**	**0.0967**	**0.1005**
	**0.0000**	**0.0000**	**0.0000**	**0.0000**
7	**0.0020**	**0.0436**	**0.1060**	**0.1192**
	**0.0000**	**0.0000**	**0.0000**	**0.0000**
8	**0.0011**	**0.0392**	**0.1121**	**0.1356**
	**0.0000**	**0.0000**	**0.0000**	**0.0000**

The first row shows the statistic associated with the test. The second row shows the p-value. Values with a p-value less than the significance level 0.05 are shown in bold. M indicates the embedding dimension and the epsilon value is determined by the expression: (0.5-2)* σ.

Table S.B4. BDS test results for GARCH (2,1) series

Épsilon/M	0.5*σ	1*σ	1.5*σ	2*σ
2	-0.0008	-0.0017	-0.0011	-0.0003
	0.0774	0.0839	0.2888	0.6243
3	-0.0004	-0.0016	-0.0011	-0.0001
	0.2219	0.1947	0.4908	0.9408
4	-0.0002	-0.0016	-0.0021	-0.0008
	0.2131	0.1503	0.3097	0.6518
5	-0.0001	-0.0010	-0.0021	-0.0014
	0.3684	0.2588	0.3470	0.5606
6	0.0000	-0.0005	-0.0019	-0.0019
	0.8951	0.4632	0.3908	0.4726
7	4 E-06	-0.0002	-0.0018	-0.0027
	0.7159	0.6754	0.3954	0.3604
8	3.2E-06	0.0000	-0.0013	-0.0029
	0.4453	0.9761	0.4853	0.3621

The first row shows the statistic associated with the test. The second row shows the p-value. Values with a p-value less than the significance level 0.05 are shown in bold. M indicates the embedding dimension and the epsilon value is determined by the expression: (0.5-2)* σ

Table S.B5. BDS test results for EGARCH (2,1) series

Épsilon/M	0.5*σ	1*σ	1.5*σ	2*σ
2	**-0.0009**	-0.0017	-0.0008	9.81E-05
	**0.0337**	0.0810	0.4250	0.8840
3	**-0.0006**	-0.0021	-0.0013	0.0002
	**0.0368**	0.0765	0.4056	0.8893
4	**-0.0003**	**-0.0022**	-0.0025	-0.0007
	**0.0216**	**0.0396**	0.1963	0.6952
5	**-0.0001**	-0.0017	-0.0028	-0.0012
	**0.0467**	0.0555	0.1938	0.6048
6	0.0000	-0.0011	-0.0026	-0.0015
	0.2024	0.1021	0.2217	0.5681
7	0.0000	-0.0006	-0.0023	-0.0019
	0.3107	0.1886	0.2518	0.5097
8	0.0000	-0.0003	-0.0016	-0.0017
	0.3684	0.3595	0.3676	0.5906

The first row shows the statistic associated with the test. The second row shows the p-value. Values with a p-value less than the significance level 0.05 are shown in bold. M indicates the embedding dimension and the epsilon value is determined by the expression: (0.5-2)* σ

B.2.2. Ibex series

Table S.B6. BDS test results for ARMA (0,3) series

Épsilon/M	0.5*σ	1*σ	1.5*σ	2*σ
2	**0.0080**	**0.0169**	**0.0161**	**0.0111**
	**0.0000**	**0.0000**	**0.0000**	**0.0000**

3	**0.0090**	**0.0327**	**0.0388**	**0.0295**
	**0.0000**	**0.0000**	**0.0000**	**0.0000**
4	**0.0069**	**0.0415**	**0.0605**	**0.0510**
	**0.0000**	**0.0000**	**0.0000**	**0.0000**
5	**0.0044**	**0.0439**	**0.0785**	**0.0734**
	**0.0000**	**0.0000**	**0.0000**	**0.0000**
6	**0.0026**	**0.0419**	**0.0908**	**0.0940**
	**0.0000**	**0.0000**	**0.0000**	**0.0000**
7	**0.0015**	**0.0381**	**0.0985**	**0.1120**
	**0.0000**	**0.0000**	**0.0000**	**0.0000**
8	**0.0008**	**0.0334**	**0.1023**	**0.1273**
	**0.0000**	**0.0000**	**0.0000**	**0.0000**

The first row shows the statistic associated with the test. The second row shows the p-value. Values with a p-value lower than the significance level 0.05 are shown in bold. M indicates the embedding dimension and the epsilon value is determined by the expression: (0.5-2)* σ

Table S.B7. BDS test results for GARCH (2,1) series

Épsilon/M	0.5*σ	1*σ	1.5*σ	2*σ
2	7.91E-06	-0.0004	-0.0004	-0.0001
	0.9827	0.6429	0.7039	0.8823
3	1.04E-05	-0.0005	-0.0008	-0.0006
	0.9654	0.6176	0.6072	0.6522
4	6.5E-05	5.2E-05	0.0000	-0.0002
	0.5826	0.9576	0.9923	0.9254
5	3.44E-05	0.0004	0.0009	0.0010
	0.4975	0.6208	0.6395	0.6598
6	1.72E-05	0.0005	0.0014	0.0016
	0.3946	0.4300	0.4693	0.5297
7	1.01E-05	0.0005	0.0018	0.0022
	0.1889	0.2505	0.3244	0.4278
8	5.3E-06	0.0003	0.0018	0.0024
	0.0616	0.2187	0.2926	0.4233

The first row shows the statistic associated with the test. The second row shows the p-value. Values with a p-value lower than the significance level 0.05 are shown in bold. M indicates the embedding dimension and the epsilon value is determined by the expression: (0.5-2)* σ.

Table S.B8. BDS test results for EGARCH (2,1) series

Épsilon/M	0.5*σ	1*σ	1.5*σ	2*σ
2	6.69E-05	-0.0001	0.0002	0.0006
	0.8492	0.9348	0.8144	0.3234
3	0.0000	-0.0003	5 E-05	0.0008
	0.9512	0.7726	0.9735	0.4992
4	2.14E-05	2.9E-06	0.0008	0.0018
	0.8496	0.9976	0.6545	0.2913
5	1.05E-05	0.0002	0.0017	0.0035
	0.8280	0.7534	0.3825	0.1103
6	1.03E-05	0.0003	0.0023	0.0048
	0.5941	0.5509	0.2384	0.0558
7	9.06E-06	0.0004	0.0027	**0.0059**

	0.2129	0.3428	0.1388	**0.0332**
8	**5.6E-06**	0.0002	0.0027	**0.0065**
	**0.0352**	0.2799	0.1122	**0.0289**

The first row shows the statistic associated with the test. The second row shows the p-value. Values with a p-value less than the significance level 0.05 are shown in bold. M indicates the embedding dimension and the epsilon value is determined by the expression: (0.5-2)* σ.

B.2.3. Nasdaq series

Table S.B9. BDS test results for ARMA (2,2) series

Épsilon/M	0.5*σ	1*σ	1.5*σ	2*σ
2	**0.0147**	**0.0260**	**0.0239**	**0.0170**
	**0.0000**	**0.0000**	**0.0000**	**0.0000**
3	**0.0178**	**0.0529**	**0.0582**	**0.0448**
	**0.0000**	**0.0000**	**0.0000**	**0.0000**
4	**0.0140**	**0.0672**	**0.0884**	**0.0742**
	**0.0000**	**0.0000**	**0.0000**	**0.0000**
5	**0.0095**	**0.0731**	**0.1144**	**0.1041**
	**0.0000**	**0.0000**	**0.0000**	**0.0000**
6	**0.0060**	**0.0728**	**0.1343**	**0.1321**
	**0.0000**	**0.0000**	**0.0000**	**0.0000**
7	**0.0037**	**0.0687**	**0.1481**	**0.1567**
	**0.0000**	**0.0000**	**0.0000**	**0.0000**
8	**0.0022**	**0.0630**	**0.1565**	**0.1780**
	**0.0000**	**0.0000**	**0.0000**	**0.0000**

The first row shows the statistic associated with the test. The second row shows the p-value. Values with a p-value less than the significance level 0.05 are shown in bold. M indicates the embedding dimension and the epsilon value is determined by the expression: (0.5-2)* σ.

Table S.B10. BDS test results for GARCH (2,1) series

Épsilon/M	0.5*σ	1*σ	1.5*σ	2*σ
2	0.0003	5.6E-05	-0.0005	-0.0004
	0.4931	0.9517	0.6156	0.5762
3	**0.0006**	0.0015	0.0008	0.0004
	**0.0265**	0.1981	0.6146	0.7679
4	**0.0003**	0.0013	0.0008	0.0004
	**0.0144**	0.2066	0.6779	0.8399
5	**0.0002**	0.0013	0.0011	0.0004
	**0.0007**	0.1179	0.5850	0.8519
6	**9.3E-05**	0.0011	0.0016	0.0007
	**0.0001**	0.0648	0.4133	0.7790
7	**4.3E-05**	**0.0010**	0.0021	0.0010
	**0.0000**	**0.0223**	0.2538	0.7074
8	**1.75E-05**	**0.0007**	0.0022	0.0010
	**0.0000**	**0.0129**	0.2001	0.7241

The first row shows the statistic associated with the test. The second row shows the p-value. Values with a p-value lower than the significance level 0.05 are shown in bold. M indicates the embedding dimension and the epsilon value is determined by the expression: (0.5-2)* σ.

Table S.B11. BDS test results for EGARCH (2,3) series

Épsilon/M	0.5*σ	1*σ	1.5*σ	2*σ
2	-0.0007	**-0.0019**	**-0.0022**	**-0.0014**
	0.0692	**0.0358**	**0.0197**	**0.0345**
3	-0.0003	-0.0018	**-0.0032**	-0.0022
	0.2052	0.0933	**0.0345**	0.0697
4	-0.0002	-0.0017	**-0.0039**	-0.0030
	0.2225	0.0925	**0.0361**	0.0842
5	0.0000	-0.0009	-0.0032	-0.0027
	0.6286	0.2530	0.1108	0.2079
6	0.0000	-0.0004	-0.0021	-0.0021
	0.9697	0.5081	0.2832	0.4122
7	6.2E-06	0.0000	-0.0011	-0.0014
	0.4451	0.9392	0.5436	0.6129
8	4.64E-06	9.13E-05	-0.0005	-0.0009
	0.1191	0.7395	0.7640	0.7631

The first row shows the statistic associated with the test. The second row shows the p-value. Values with a p-value lower than the significance level 0.05 are shown in bold. M indicates the embedding dimension and the epsilon value is determined by the expression: (0.5-2) σ.

B.2.4. Nikkei series

Table S.B12. BDS test results for ARMA (0,1) series

Épsilon/M	0.5*σ	1*σ	1.5*σ	2*σ
2	0.0037	0.0093	0.0111	0.0090
	0.0000	0.0000	0.0000	0.0000
3	0.0043	0.0182	0.0263	0.0233
	0.0000	0.0000	0.0000	0.0000
4	0.0031	0.0222	0.0393	0.0384
	0.0000	0.0000	0.0000	0.0000
5	0.0020	0.0225	0.0488	0.0527
	0.0000	0.0000	0.0000	0.0000
6	0.0011	0.0210	0.0561	0.0672
	0.0000	0.0000	0.0000	0.0000
7	0.0006	0.0185	0.0607	0.0802
	0.0000	0.0000	0.0000	0.0000
8	0.0003	0.0158	0.0632	0.0917
	0.0000	0.0000	0.0000	0.0000

The first row shows the statistic associated with the test. The second row shows the p-value. Values with a p-value less than the significance level 0.05 are shown in bold. M indicates the embedding dimension and the epsilon value is determined by the expression: (0.5-2)*σ.

Table S.B13. BDS test results for GARCH(1,1) series

Épsilon/M	0.5*σ	1*σ	1.5*σ	2*σ
2	**-0.0014**	**-0.0036**	**-0.0033**	**-0.0016**
	**0.0004**	**0.0001**	**0.0006**	**0.0134**

3	**-0.0007**	**-0.0037**	**-0.0045**	**-0.0026**
	**0.0083**	**0.0012**	**0.0038**	**0.0359**
4	**-0.0003**	**-0.0030**	**-0.0051**	-0.0034
	**0.0322**	**0.0045**	**0.0087**	0.0557
5	-0.0001	**-0.0021**	**-0.0052**	-0.0043
	0.0611	**0.0110**	**0.0116**	0.0539
6	0.0000	**-0.0013**	**-0.0046**	-0.0043
	0.1708	**0.0375**	**0.0265**	0.0995
7	0.0000	-0.0007	**-0.0038**	-0.0040
	0.4401	0.0887	**0.0484**	0.1607
8	0.0000	-0.0004	-0.0029	-0.0032
	0.9793	0.2203	0.1070	0.2897

The first row shows the statistic associated with the test. The second row shows the p-value. Values with a p-value lower than the significance level 0.05 are shown in bold. M indicates the embedding dimension and the epsilon value is determined by the expression: (0.5-2)*σ.

Table S.B14. BDS test results for EGARCH (1,1) series

**Épsilon/M**	**0.5*σ**	**1*σ**	**1.5*σ**	**2*σ**
2	**-0.0017**	**-0.0044**	**-0.0041**	**-0.0020**
	**0.0000**	**0.0000**	**0.0000**	**0.0019**
3	**-0.0010**	**-0.0049**	**-0.0063**	**-0.0035**
	**0.0001**	**0.0000**	**0.0001**	**0.0044**
4	**-0.0005**	**-0.0043**	**-0.0075**	**-0.0048**
	**0.0002**	**0.0000**	**0.0001**	**0.0074**
5	**-0.0002**	**-0.0032**	**-0.0079**	**-0.0060**
	**0.0008**	**0.0001**	**0.0001**	**0.0074**
6	**-0.0001**	**-0.0021**	**-0.0072**	**-0.0060**
	**0.0054**	**0.0004**	**0.0004**	**0.0189**
7	**0.0000**	**-0.0014**	**-0.0063**	**-0.0057**
	**0.0259**	**0.0013**	**0.0010**	**0.0432**
8	**0.0000**	**-0.0008**	**-0.0050**	**-0.0048**
	**0.1230**	**0.0057**	**0.0036**	**0.1079**

The first row shows the statistic associated with the test. The second row shows the p-value. Values with a p-value lower than the significance level 0.05 are shown in bold. M indicates the embedding dimension and the epsilon value is determined by the expression: (0.5-2)*σ.

B.3. Results of Kaplan's test

B.3.1. Dow Jones

Table S.B15. Results of Kaplan's test for Dow Jones series

	Dim	Mean K	Standard Deviation	Min	K	K*
**Returns**	1	0.0129	0.0013	0.0025	**0.0104**	1.2495
	2	0.0121	0.0013	0.0026	**0.0108**	1.1239
	3	0.0130	0.0025	0.0050	**0.0084**	1.5465
	4	0.0128	0.0019	0.0039	**0.0073**	1.7509
	5	0.0122	0.0029	0.0013	**0.0047**	2.5802
	6	0.0119	0.0033	-0.0018	**0.0068**	1.7598
	7	0.0112	0.0036	0.0032	**0.0141**	0.7991
	8	0.0123	0.0046	-0.0085	**0.0043**	2.8763
**ARMA (2,5)**	1	0.0129	0.0010	0.0019	**0.0113**	1.1359
	2	0.0123	0.0024	0.0005	**0.0084**	1.4557
	3	0.0114	0.0054	-0.0097	**0.0082**	1.3783
	4	0.0130	0.0026	0.0052	**0.0067**	1.9424
	5	0.0116	0.0071	-0.0247	**0.0059**	1.9530
	6	0.0115	0.0051	-0.0139	**0.0088**	1.3120
	7	0.0145	0.0070	0.0068	**0.0067**	2.1776
	8	0.0124	0.0036	-0.0044	**0.0052**	2.4116
**GARCH (2,1)**	1	1.1428	0.0653	0.1306	**1.1142**	1.0257
	2	1.1335	0.1163	0.2327	**1.2234**	0.9265
	3	1.0958	0.1338	0.2677	**1.0798**	1.0148
	4	1.0778	0.1776	0.3552	**1.1901**	0.9056
	5	1.1041	0.1572	0.3144	**1.0715**	1.0304
	6	1.0359	0.3257	0.1600	**1.0760**	0.9627
	7	1.2312	0.2927	0.5854	**1.0400**	1.1838
	8	1.2915	1.3532	-1.2372	**0.9974**	1.2949
**EGARCH (2,1)***	1	1.1101	0.1326	0.6392	**1.1197**	0.9914
	2	1.1461	0.1089	0.8287	**1.0954**	1.0463
	3	1.1171	0.1351	0.5923	**1.2287**	0.9091
	4	1.1529	0.1220	0.9110	**1.0583**	1.0894
	5	0.8925	0.6679	-2.1912	**1.0710**	0.8334
	6	1.1824	0.1727	0.7701	**1.1038**	1.0712
	7	1.0948	0.2777	-0.0177	**1.1928**	0.9179
	8	1.0248	0.7234	-2.5085	**1.5416**	0.6648

Results of the Kaplan test. on 30 substitute data for the series Returns. ARMA (2,5). GARCH (2,1) and EGARCH (2,1) The value of K* has been calculated as the quotient between the mean and K. the statistic. The minimum is the smallest value between the minimum of replications and the mean minus 2 times the standard deviation. K. Dim represents the embedding dimension and corresponds to the m-1 dimension of the BDS test. * The method of the first minimum of the mutual information function determined that the optimal value of delay was t=2. The analyses were repeated considering t=2 obtaining the same results.

B.3.2. Ibex 35

Table S.B16. Results of Kaplan's test for Ibex series

	Dim	Mean K	Standard Deviation	Min	K	K*
**Returns**	1	0.0162	0.0010	0.0019	**0.0140**	1.1626
	2	0.0174	0.0114	0.0024	**0.0115**	1.5147
	3	0.0166	0.0033	0.0066	**0.0122**	1.3630
	4	0.0159	0.0028	0.0057	**0.0086**	1.8584
	5	0.0172	0.0034	0.0067	**0.0028**	6.2194
	6	0.0165	0.0037	0.0027	**0.0102**	1.6280
	7	0.0166	0.0041	0.0079	**0.0101**	1.6441
	8	0.0148	0.0039	0.0037	-0.0012	-12.700
**ARMA (0,3)**	1	0.0161	0.0011	0.0021	**0.0139**	1.1559
	2	0.0163	0.0019	0.0037	**0.0133**	1.2182
	3	0.0163	0.0019	0.0039	**0.0111**	1.4731
	4	0.0161	0.0035	0.0005	**0.0157**	1.0260
	5	0.0174	0.0062	0.0061	0.0034	5.1566
	6	0.0181	0.0042	0.0083	**0.0090**	2.0049
	7	0.0165	0.0034	0.0058	0.0031	5.2801
	8	0.0167	0.0032	0.0064	-0.0026	-6.5282
**GARCH (2,1)***	1	1.1173	0.0552	0.1104	**1.0686**	1.0456
	2	1.1643	0.1001	0.2002	**1.2538**	0.9286
	3	1.1680	0.1827	0.3655	**1.1486**	1.0169
	4	1.0985	0.1695	0.3390	**0.6817**	1.6114
	5	1.0875	0.2889	0.0224	**1.0519**	1.0338
	6	1.1662	0.2609	0.5219	**1.0159**	1.1479
	7	1.1756	0.2724	0.5107	**1.0062**	1.1684
	8	1.3800	1.0024	0.3248	**1.0620**	1.2994
**EGARCH (2,1)**	1	1.1221	0.0547	0.1093	**1.0066**	1.1148
	2	1.1442	0.1366	0.2731	**1.1400**	1.0036
	3	1.0872	0.1706	0.3413	**0.9222**	1.1789
	4	1.1827	0.2557	0.4947	0.4432	2.6685
	5	1.1451	0.1926	0.3853	**1.0199**	1.1228
	6	1.1542	0.1966	0.3932	**1.0221**	1.1293
	7	1.1392	0.2477	0.4954	**0.9926**	1.1476
	8	1.2046	0.2567	0.5134	**0.9564**	1.2596

Results of the Kaplan test. on 30 substitute data for the series Returns. ARMA (0,3). GARCH (2,1) and EGARCH (2,1). The value of K* has been calculated as the quotient between the mean and K, the statistic. The minimum is the smallest value between the minimum of replications and the mean minus 2 times the standard deviation. K. Dim represents the embedding dimension and corresponds to the m-1 dimension of the BDS test. * The method of the first minimum of the mutual information function determined that the optimal value of delay was t=2. The analyses were repeated considering t=2 obtaining the same results.

B.3.3. Nasdaq

Table S.B17. Results of Kaplan's test for Nasdaq series

	Dim	Mean K	Standard Deviation	Min	K	K*
**Returns**	1	0.0177	0.0014	**0.0147**	0.0132	1.3447
	2	0.0180	0.0016	**0.0146**	0.0117	1.5426
	3	0.0171	0.0014	**0.0126**	0.0102	1.6694
	4	0.0199	0.0037	**0.0145**	0.0096	2.0636
	5	0.0174	0.0027	**0.0116**	0.0089	1.9690
	6	0.0176	0.0023	**0.0125**	0.0072	2.4568
	7	0.0181	0.0041	**0.0113**	0.0080	2.2666
	8	0.0188	0.0039	0.0058	**0.0074**	2.5389
**ARMA (2,2)**	1	0.0176	0.0011	0.0019	**0.0022**	1.1918
	2	0.0184	0.0032	0.0005	**0.0065**	1.6788
	3	0.0173	0.0037	-0.0097	**0.0032**	1.7360
	4	0.0099	0.0442	**0.0052**	-0.2235	1.0744
	5	0.0173	0.0040	-0.0247	**0.0058**	2.3410
	6	0.0153	0.0162	**-0.0139**	-0.0692	2.0146
	7	0.0185	0.0057	0.0068	**0.0104**	2.2455
	8	0.0170	0.0035	-0.0044	**0.0069**	2.5824
**GARCH (2,1)***	1	1.2268	0.5096	1.0184	**1.0759**	1.1403
	2	1.1042	0.2180	0.1866	**1.0204**	1.0821
	3	1.1101	0.1068	0.2136	**1.0544**	1.0528
	4	1.0958	0.1318	0.2636	**0.9000**	1.2176
	5	1.1607	0.1942	0.3885	**1.2814**	0.9058
	6	1.1051	0.1686	0.3372	**1.0004**	1.1046
	7	0.9678	1.2571	-5.6163	**0.9382**	1.0316
	8	1.1199	0.2944	0.2773	**0.9683**	1.1565
**EGARCH (2,3)***	1	1.1191	0.0587	0.1175	**1.0425**	1.0734
	2	1.0922	0.1077	0.2154	**1.2279**	0.8894
	3	1.1759	0.3110	0.6220	**1.1221**	1.0479
	4	1.1137	0.1252	0.2504	**1.2064**	0.9232
	5	1.9263	4.2907	0.8193	**1.1256**	1.7114
	6	1.1506	0.3847	-0.0712	**0.9927**	1.1591
	7	1.1572	0.2365	0.3746	**1.1420**	1.0133
	8	1.0365	0.6453	-1.6612	**1.0718**	0.9670

Results of the Kaplan's test. on 30 substitute data for the series Returns. ARMA (2,2). GARCH (2,1) and EGARCH (2,3) The value of K* has been calculated as the quotient between the mean and K. the statistic. The minimum is the smallest value between the minimum of replications and the mean minus 2 times the standard deviation. K. Dim represents the embedding dimension and corresponds to the m-1 dimension of the BDS test. * The method of the first minimum of the mutual information function determined that the optimum value of delay was t=2 and t=3 for the GARCH (2,1) and EGARCH (2,3) series respectively. The analyses were repeated considering the optimal delay values. obtaining the same results.

B.3.4. Nikkei

Table S.B18. Results of Kaplan's test for Nikkei series

	Dim	Mean K	Standard Deviation	Mínimum	K	K*
**Returns**	1	0.0169	0.0015	0.0030	**0.0144**	1.1730
	2	0.0172	0.0016	0.0031	**0.0130**	1.3252
	3	0.0174	0.0024	0.0049	**0.0116**	1.4925
	4	0.0174	0.0028	0.0056	**0.0118**	1.4728
	5	0.0179	0.0023	0.0046	**0.0119**	1.5103
	6	0.0169	0.0034	0.0068	**0.0101**	1.6705
	7	0.0150	0.0169	-0.0726	**0.0086**	1.7514
	8	0.0167	0.0080	-0.0101	**0.0067**	2.4879
**ARMA (0,1)**	1	0.0170	0.0019	0.0097	**0.0157**	1.0793
	2	0.0170	0.0016	0.0127	**0.0148**	1.1516
	3	0.0165	0.0047	-0.0031	**0.0126**	1.3045
	4	0.0173	0.0015	**0.0142**	0.0110	1.5680
	5	0.0165	0.0034	0.0049	**0.0119**	1.3895
	6	0.0163	0.0039	0.0068	**0.0100**	1.6357
	7	0.0163	0.0029	0.0086	**0.0092**	1.7663
	8	0.0154	0.0082	-0.0123	**0.0068**	2.2635
**GARCH (1,1)***	1	1.1199	0.0992	0.1983	**1.2176**	0.9198
	2	1.1586	0.3144	0.6288	**1.1099**	1.0439
	3	0.9567	1.0579	-4.5799	**1.1550**	0.8283
	4	1.1139	0.1715	0.3431	**1.1342**	0.9821
	5	1.1972	0.5010	0.4110	**0.8859**	1.3513
	6	1.2555	0.7925	**-0.0190**	-0.0266	-47.211
	7	1.1724	0.8407	-0.7760	**1.0556**	1.1106
	8	1.0854	0.4108	-0.1333	**1.0384**	1.0453
**EGARCH (1,1)***	1	1.1255	0.0692	0.1384	**1.1603**	1.1603
	2	1.1327	0.1118	0.2235	**1.1887**	1.1887
	3	1.0636	0.3138	-0.4517	**1.1451**	1.1451
	4	1.1180	0.1646	0.3292	**0.6262**	0.6262
	5	0.9025	0.8652	-3.4825	**1.0557**	1.0557
	6	1.1616	0.3745	0.0305	**0.6367**	0.6367
	7	1.1339	0.3865	-0.1101	**0.7754**	0.7754
	8	1.1401	0.1258	0.2516	**1.1814**	1.1814

Results of the Kaplan test on 30 substitute data for the series Returns. ARMA(0,1). GARCH (1,1) and EGARCH (1,1). The value of K* has been calculated as the quotient between the mean and K. the statistic. The minimum is the smallest value between the minimum of replications and the mean minus 2 times the standard deviation. K. Dim represents the embedding dimension and corresponds to the m-1 dimension of the BDS test. * The method of the first minimum of the mutual information function determined that the optimum value of delay was t=3 and t=5 for the GARCH(1,1) and EGARCH(1,1) series respectively. The analyses were repeated considering the optimal delay values. obtaining the same result.

C: Chaotic Component Study

C.1. Correlation Dimension Results

Table S.C1. Correlation Dimension

Dow Jones				
**M**	**Returns^{1}**	**ARMA (2,5)^{1}**	**GARCH (2,1)^{2}**	**EGARCH (2,1)^{1}**
1	0.90	0.91	0.98	0.98
2	1.78	1.80	1.96	1.96
3	2.62	2.66	2.94	2.94
4	3.45	3.51	3.95	3.92
5	4.24	4.32	5.01	5.53
6	5.00	5.10	5.92	5.75
7	5.75	5.86	6.54	5.00
8	6.49	6.59	7.32	5.17
Ibex 35				
**M**	**Returns^{1}**	**ARMA (0,3)^{1}**	**GARCH (2,1)^{1}**	**EGARCH (2,1)^{2}**
1	0.93	0.93	0.97	0.97
2	1.84	1.84	1.95	1.95
3	2.72	2.72	2.92	2.91
4	3.56	3.57	3.86	3.87
5	4.35	4.40	4.82	4.83
6	5.05	5.19	5.74	5.70
7	5.98	5.96	6.58	6.58
8	6.52	6.65	7.10	7.29
Nasdaq				
**M**	**Returns^{1}**	**ARMA (2,2)^{1}**	**GARCH (2,1)^{3}**	**EGARCH (2,3)^{5}**
1	0.91	0.91	0.98	0.98
2	1.78	1.79	1.96	1.97
3	2.61	2.63	2.93	2.95
4	3.42	3.45	3.91	3.91
5	4.21	4.25	4.79	4.93
6	5.00	5.04	5.72	5.75
7	5.77	5.83	6.97	6.62
8	6.53	6.62	7.49	6.99
Nikkei				
**M**	**Returns^{1}**	**ARMA (0,1)^{1}**	**GARCH (1,1)^{3}**	**EGARCH (1,1)^{2}**
1	0.93	0.93	0.97	0.97
2	1.86	1.86	1.95	1.94
3	2.77	2.78	2.92	2.91
4	3.66	3.68	3.88	3.88
5	4.52	4.54	4.88	4.89
6	5.34	5.36	5.89	6.02
7	6.13	6.17	7.08	7.16
8	6.87	6.93	8.37	8.45

Next to each applied model. the optimal value of the delay time according to the criterion of minimum information is indicated as a superscript. M represents the embedding value of the series.

C.2. Lyapunov test results

Table S.C2. Lyapunov test results

	(L.m.q)	λ	p-value*	*Hypothesis*
Dow Jones				
Returns	(2.6.3)	-0.1701	0.000	H_1
ARMA (2,5)	(3.6.3)	-0.4954	0.000	H_1
GARCH (2,1)	(2.6.1)	-0.6163	0.000	H_1
EGARCH (2,1)	(2.6.3)	-0.6903	0.000	H_1
Ibex 35				
Returns	(4.6.2)	-0.1725	0.000	H_1
ARMA (0,3)	(4.6.4)	-0.4111	0.000	H_1
GARCH (2,1)	(5.6.4)	-0.4968	0.000	H_1
EGARCH (2,1)	(3.6.3)	-0.5705	1.36E-17	H_1
Nasdaq				
Returns	(3.6.3)	-0.4247	0.00E+00	H_1
ARMA (2,2)	(2.6.4)	-0.4001	0.00E+00	H_1
GARCH (2,1)	(2.6.5)	-0.4803	1.33E-252	H_1
EGARCH (2,2)	(5.6.4)	-0.6798	1.60E-173	H_1
Nikkei				
Returns	(4.6.2)	-0.5386	0.000	H_1
ARMA (0,1)	(4.6.3)	-0.5363	0.000	H_1
GARCH (1,1)	(2.5.2)	-0.6657	1.14E-283	H_1
EGARCH (1,1)	(2.5.4)	-0.6897	5.31E-123	H_1

At 5% significance level, the Null hypothesis of the existence of a chaotic component is rejected for those p-values less than 0.05.

C.3. Results of the 0/1 test and the Hurst Exponent

Table S.C3. Results of the 0/1 test

	0/1 test
Dow Jones	
Returns	0.9980
ARMA (2,5)	0.9978
GARCH (2,1)	0.9983
EGARCH (2,1)	0.9982
Ibex 35	
Returns	0.9979
ARMA (0,3)	0.9981
GARCH (2,1)	0.9982
EGARCH (2,1)	0.9981
Nasdaq	
Returns	0.9977
ARMA (2,2)	0.9977
GARCH (2,1)	0.9982
EGARCH (2,3)	0.9982

Nikkei	
Returns	0.9980
ARMA (0,1)	0.9981
GARCH (1,1)	0.9982
EGARCH (1,1)	0.9982

A total of 6000 points were used for the 0-1 method. * Results were significant at a 5% confidence level. (H) corresponds to the value of the Hurst coefficient.

C.4. MGRM test results

Table S.C4. MGRM test results

	**Exponent (α)**	**Standard Deviation**
Dow Jones		
GARCH(2,1)	0.2730	0.0094
EGARCH(2,1)	0.3034	0.0123
Ibex 35		
GARCH(2,1)	0.2260	0.0054
EGARCH(2,1)	0.2537	0.0077
Nasdaq		
GARCH(2,1)	0.2456	0.0091
EGARCH(2,3)	0.2290	0.0089
Nikkei		
GARCH(1,1)	0.2300	0.0102
EGARCH(1,1)	0.2450	0.0089

To perform the test the parameter *w* was set to four.
